# Negative effects of short birth interval on child mortality in low- and middle-income countries: A systematic review and meta-analysis

**DOI:** 10.7189/jogh.12.04070

**Published:** 2022-09-03

**Authors:** Mohammad Zahidul Islam, Arif Billah, M Mofizul Islam, Mostafizur Rahman, Nuruzzaman Khan

**Affiliations:** 1 Department of Population Science, Jatiya Kabi Kazi Nazrul Islam University, Mymensingh, Bangladesh; 2 Department of Population Science and Human Resource Development, University of Rajshahi, Bangladesh; 3 Department of Social Work and Counselling, Faculty of Business, Economics and Social Development, Universiti Malaysia Terengganu, Malaysia; 4 Department of Public Health, La Trobe University, Bundoora, Victoria, Australia; **Background** Short birth interval (SBI) is linked with higher rates of child mortality in low- and lower-middle-income countries (LMICs). In this study, we estimated the summary effects of SBI on several forms of child mortality in LMICs.

## Abstract

**Methods:**

Eight databases, PubMed, CINAHL, Web of Science, Embase, PsycINFO, Cochrane Library, Popline, and Maternity and Infant Care, were searched, covering the period of January 2000 to January 2022. Studies that had examined the association between SBI and any form of child mortality were included. The findings of the included studies were summarized through fixed-effects or random-effects meta-analysis and the model was selected based on the heterogeneity index.

**Results:**

A total of 51 studies were included. Of them, 19 were conducted in Ethiopia, 10 in Nigeria and 7 in Bangladesh. Significant higher likelihoods of stillbirth (odds ratio (OR) = 2.11; 95% confidence interval (CI) = 1.32-3.38), early neonatal mortality (OR = 1.58; 95% CI = 1.04-2.41), perinatal mortality (OR = 1.71; 95% CI = 1.32-2.21), neonatal mortality (OR = 1.85; 95% CI = 1.68-2.04), post-neonatal mortality (OR = 3.01; 95% CI = 1.43-6.33), infant mortality (OR = 1.92; 95% CI = 1.77-2.07), child mortality (OR = 1.67; 95% CI = 1.27-2.19) and under-five mortality (OR = 1.95; 95% CI = 1.56-2.44) were found among babies born in short birth intervals than those who born in normal intervals.

**Conclusions:**

SBI significantly increases the risk of child mortality in LMICs. Programmes to reduce pregnancies in short intervals need to be expanded and strengthened. Reproductive health interventions aimed at reducing child mortality should include proper counselling on family planning, distribution of appropriate contraceptives and increased awareness of the adverse effects of SBI on maternal and child health.

Currently, the global neonatal mortality rate is 17 per 1000 live births and under-five mortality (U5M) is 37 per 1000 live births [1,2]. These rates declined substantially in the Millennium Development Goal period, 2000-2015 [3]. The Sustainable Development Goal targets to reduce neonatal and U5M to less than 12 and 25 per 1000 live births, respectively [3]. A majority of these deaths occur in low- and lower-middle-income countries (LMICs), where the neonatal mortality and U5M rates are around 10 and 15 times higher than in high-income countries [4,5]. The deaths rates are even higher among the children of their first month of life. Over 46% of the 5.2 million U5M recorded in 2019 occurred in the first month of life, around 33% occurred on the first day of birth and close to 75% within the first week of birth [3]. The most common causes of these deaths are complications during pregnancy as well as adverse birth outcomes including premature birth, low birth weight and birth defects [6]. Significant progress can be made in reducing these deaths by offering appropriate maternal health care services.

The World Health Organization (WHO) recommends an optimal time-space between two successive births, 24 months for two subsequent conceptions and 33 months for one conception to the next live birth. Shorter than these recommended durations are identified as short birth intervals (SBIs). Between 11% and 66% of all live births in LMICs occur in short intervals and contribute to several adverse pregnancy and birth outcomes, including maternal and under-five mortality [7-11]. Possible pathways of such contributions are both direct and indirect. For instance, mothers giving birth in SBIs are usually undernourished and anemic, which, in turn, contribute to increased risks of pregnancy complications and maternal mortality. SBIs increase the risks of a range of adverse outcomes of child health, including preterm birth, under-nutrition and low-birth-weight [6,12], and these outcomes subsequently contribute to U5M in several LMICs [13,14].

Relevant studies exploring these associations were conducted mostly in African contexts, although the prevalence of SBI is higher in Asian countries (33%) than in African countries (20%) [7]. Moreover, maternal health care services use, which can significantly reduce adverse pregnancy and birth outcomes, are substantially different in LMICs, with higher rates in Asian countries and lower in African countries [15,16]. Thus, the observed associations in African countries may not be generalisable in other LMICs. Moreover, studies conducted in the context of African countries with regard to SBI examined mainly the relationship with U5M [10,17,18] but rarely with other forms of child mortality, such as neonatal mortality and infant mortality. The relationships of SBIs are not uniform across these various forms of child mortality. For instance, the negative effect of SBI was found stronger for neonatal mortality than infant mortality [19]. Having a detailed picture of the relationships between SBIs and various forms of child mortality such as stillbirth, neonatal mortality, post-natal mortality, infant mortality in the context of LMICs generated from a systematic review may help overcome some of these limitations, offer an overall overview. Such a study would inform with detailed risks of SBIs on the child mortality across the respective durations of each of these health outcomes and assist in evidence-based policy and programmes. However, systematic reviews that have been conducted so far examined the effects of SBI on low-birth-weight, preterm births and stillbirths [6,20-23]. Also, the individual studies around the effects of SBI on different forms of child mortality are scarce in the context of LMICs and available studies are country-specific [24]. Consequently, the summary effects of SBI on several form of child mortality in LMICs are still unknown. We therefore conducted this study to determine the effects of SBI on various forms of child mortality in LMICs.

## METHODS

We conducted a systematic review and meta-analysis and reported the findings following the Preferred Reporting Items of Systematic Review and Meta-analysis (PRISMA) consensus statement on the conduct of meta-analysis of observational studies. Relevant and available studies about the relationship between SBIs and various forms of child mortality were included.

### Search strategy

A systematic literature search was conducted in January 2022 in the eight databases: PubMed, Cumulative Index to Nursing & Allied Health (CINAHL), Web of Science, Embase, PsycINFO, Cochrane Library, Popline, and Maternity and Infant Care. Studies published since the establishment of the MDGs from January 2000 to January 2022 were included. Searches were conducted on the basis of the individual comprehensive search strategies for each database. We developed search strategies using a combination of free text words, words in title/abstracts and medical subject headings (MeSH) terms of exposure, outcomes and settings. The exposure-related terms were birth intervals or short birth intervals or pregnancy intervals. The outcome-related terms were stillbirths or early neonatal mortality or perinatal mortality or neonatal mortality or post-neonatal mortality or infant mortality or child mortality or under-five mortality. Finally, LMICs’ names were included as the terms related to study settings. They are Afghanistan, Angola, Armenia, Bangladesh, Benin, Bhutan, Bolivia, Burkina Faso, Burundi, Cabo Verde, Cambodia, Cameroon, Central African Republic, Chad, Comoros, Congo, Cote d'Ivoire, Djibouti, Egypt, El Salvador, Eritrea, Ethiopia, Gambia, Georgia, Ghana, Guatemala, Guinea-Bissau, Haiti, Honduras, India, Indonesia, Jordan, Kenya, Kiribati, Korea, Kosovo, Kyrgyz Republic, Laos, Lesotho, Liberia, Madagascar, Malawi, Mali, Mauritania, Micronesia, Moldova, Mongolia, Morocco, Mozambique, Myanmar, Namibia, Nepal, Nicaragua, Niger, Nigeria, Pakistan, Papua New Guinea, Philippine, Rwanda, Sao Tome & Principe, Senegal, Sierra Leone, Solomon Island, Somalia, South Sudan, Sri Lanka, Sudan, Swaziland, Syria, Tajikistan, Tanzania, Timor-Leste, Togo, Tunisia, Uganda, Ukraine, Uzbekistan, Vanuatu, Vietnam, West Bank and Gaza, Yemen, Zambia, Zimbabwe. For this classification, we followed the 2021 World Bank country classification [25]. The term LMICs was also considered together with country names. We combined these terms using the Boolean operators (AND, OR). The Full search strategy and search results for each database are presented in Tables S1 to S8 in the **Online Supplementary Document**. Further searches for eligible studies were conducted by reviewing selected journals’ websites and reference lists of the selected articles.

### Study selection

Two authors (MZI and MAB) independently reviewed all articles based on the inclusion and exclusion criteria presented in **Table 1**. They first conducted title and abstract screening. Articles selected in this stage were considered for full-text review. Disagreements were resolved through discussion and involving the senior author (MNK) if deemed necessary. We used online platforms, including COVIDENCE, EndNote X9 and Zoom online meetings to complete this review.

**Table 1:** Inclusion and exclusion criteria considered in this study

Topics	Criteria for inclusion	Criteria for exclusion
Study type	Peer-reviewed	Non-peer reviewed
Language	In English only	Other than English language
Exposure	Short birth interval	
Outcome	Any form of child mortality including stillbirths, neonatal mortality, perinatal mortality and under-5 mortality (U5M)	Other than the child mortality
Country type	Low and lower-middle income countries	Higher-middle- and high-income countries
Participant	Women with pregnancy outcomes	Women with or without pregnancy outcome and have any suppressible conditions – eg, HIV/AIDS or other STI infections, having chronic non-communicable diseases

### Data extraction

Prior to tabulating the final data, a data extraction template was designed, trialled, and modified following the “Strengthening the Reporting of Observational Studies in Epidemiology” guidelines [26]. Two authors (MZI and MAB) independently extracted the relevant data, including authors’ names, design of the study, study sample size, study setting, and the category(ies) of child mortality. Other information collected were effects size (eg, odds ratio (OR)) and underlying data used to calculate it and whether it was adjusted or unadjusted for confounders. Similar to the previous step, disagreements between the data collectors were resolved through discussion and involving the senior author (MNK) when deemed necessary.

### Quality assessment of included studies

We used the modified Newcastle-Ottawa Scale (NOS) for the quality assessment of the included studies [27]. The items included in the scale were different for the cross-sectional, case-control and cohort studies. Two authors (MZI and MAB) checked included articles and gave 1 point for each item if the study met the relevant item condition. Aggregated scores were then used to measure overall study quality as good (score 8 to 9), moderate (score 5 to 7) and low (score <5) [28,29].

### Exposure variable

Our exposure of interest was SBI, classified dichotomously as Yes and No. Some articles included in this review used slightly different intervals to define SBI. However, for the quantitative synthesis, we followed the WHO classification of SBI [30]. The articles that did not follow the WHO classification were synthesized narratively.

### Outcome variables

The following seven forms of child mortality were our outcome variables: stillbirth (a baby dies after 28 weeks of pregnancy, but before or during birth), early neonatal mortality (death between 0 and 7 completed days of birth), perinatal death (death within 28 weeks of gestation to one week of live birth), neonatal death (deaths among live births during the first 28 completed days of life), post-neonatal mortality (death after 7 days to 28 completed days of birth), infant death (death before completing the first year of age), child mortality (death between the first and fifth birthday) and U5M (death after birth, but before reaching the age of five years) [31-36].

### Statistical analysis

We used extracted ORs as the basis of analysis. If ORs were unavailable in the paper, we first calculated unadjusted ORs. For the studies conducted in multiple countries and that reported multiple ORs, we computed pooled ORs first. We used the fixed-effects and random-effects models to calculate the summary estimate for the overall effects of SBI on stillbirth, early neonatal mortality, perinatal mortality, neonatal mortality, post-neonatal mortality, infant mortality, child mortality and U5M. We did not reclassify these mortality measures. For instance, both neonatal mortality and post-neonatal mortality are parts of infant mortality and they could be pooled together to get the summary estimate for infant mortality. The model was selected based on the heterogeneity assessment. An I^2^ statistic with a *p*-value (<0.05) was estimated for each meta-analysis to describe the extent of heterogeneity. When the test for heterogeneity was moderate (50%-74%) or high (75%-100%), the pooled estimates of ORs were computed by using the random-effects model [37]. We also explored the source of heterogeneity using subgroup analysis and meta-regression once moderate or higher heterogeneity was identified. For this, pre-specified sub-groups were considered including the study sample size, confounding adjustment, study design, and study settings. Publication bias was explored by visual inspection of Funnel Plot asymmetry and Egger’s regression test [38]. The trim-and-fill method was used when evidence of publication bias was found, and to estimate and adjust for potentially missing studies, the effect size was recalculated accordingly [39]. Statistical software, STATA version 15.1 (Stata.corp), was used for all analyses.

## RESULTS

### Search results

**Figure 1:**
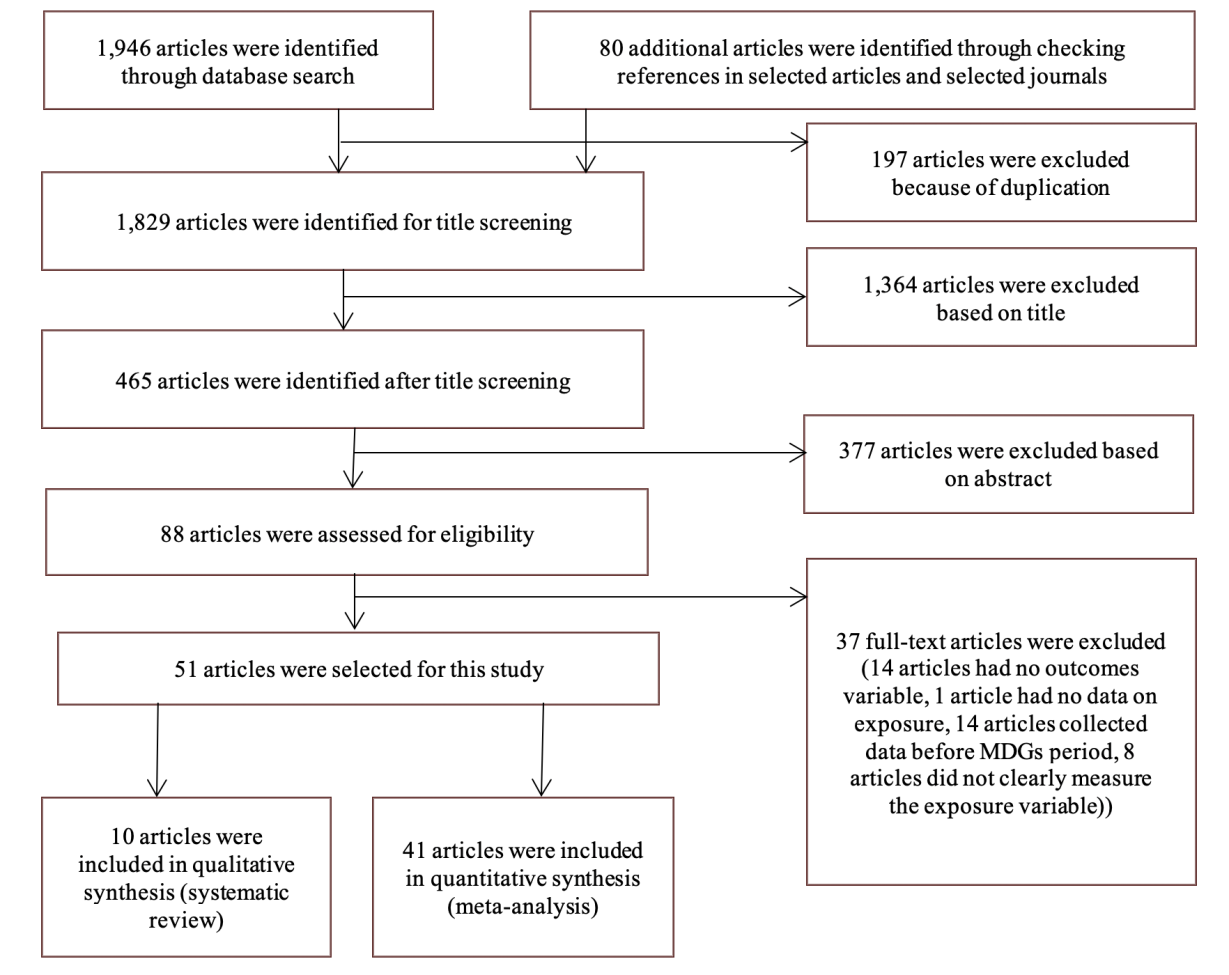
Schematic presentation of the studies included and excluded in the systematic review.

We found 1946 studies in eight databases and additional 80 studies through hand searching and reference checking. Together, there were 2026 studies, of which 197 studies were duplicates and thereby excluded. Of the remaining 1829 studies, 1741 studies were excluded through title and abstract screening. Full-text reviews were conducted for the remaining 88 articles and 37 articles were excluded based on full-text review. We excluded those 37 studies because they did not report any mortality outcome (n = 14), or collected data before the starting point of MDGs (ie, prior to January 2000) (n = 14), or did not clearly report the measurement criteria (n = 8) or did not have any data on exposure variable (n = 1). A total of 51 studies were finally included in this study, 41 included in the quantitative synthesis and the remaining 10 studies included in the narrative synthesis **(****Figure 1**). They were moderate to good in quality (see Tables S10-S12 in the **Online Supplementary Document**).

### Study characteristics

Of the 51 studies included, a majority were conducted in Ethiopia (n = 19) [40-58], Nigeria (n = 10) [17,59-67] and Bangladesh (n = 7) [68-74] (Table S9 in the **Online Supplementary Document**). Four studies were conducted in multiple countries [17,56,57,70]. Forty-five of the included studies were of cross-sectional design. More than two-thirds (n = 36) of the included studies used national-level data and fourteen studies reported adjusted ORs.

### Relationship between short birth interval on child mortality

We found significantly higher likelihoods of stillbirth, early neonatal mortality, perinatal mortality, neonatal mortality, post-neonatal mortality, infant mortality, child mortality and U5M among children of mothers having SBI than their counterpart mothers having optimal birth intervals (**Table 2**).

**Table 2:** Summary measures of the relationships between short birth interval and various forms of child mortality, publication bias, and Trim and Fill estimates for low- and lower-middle-income countries, January 2000 to January 2022

Characteristics	Number of studies	Summary estimates *		Egger bias test *P*-value	Trim and Fill estimates ^†^	
**OR (95%, CI)**	**Heterogeneity index**	**Number of missing studies**	**OR (95%, CI)**
**Adverse outcomes**						
Stillbirth	4	2.11 (1.32-3.38)	76.0%	0.651	0	2.11 (1.32-3.38)
Early neonatal mortality	1	1.58 (1.04-2.41)	0.0%	0.422	0	1.58 (1.04-2.41)
Perinatal mortality	4	1.71 (1.32-2.21)	76.5%	0.090	0	1.71 (1.32-2.21)
Neonatal mortality	17	1.85 (1.68-2.04)	72.6%	<0.01	3	1.76 (1.60-1.95)
Post-neonatal mortality	1	3.01 (1.43-6.33)	0.00%	0.428	0	3.01 (1.43-6.33)
Infant mortality	12	1.92 (1.77-2.07)	51.4%	<0.01	2	1.88 (1.73-2.04)
Child mortality	9	1.67 (1.27-2.19)	89.3%	<0.01	2	1.44 (1.09-1.90)
Under-five mortality	9	1.95 (1.56-2.44)	90.3%	<0.01	1	1.89 (1.52-2.34)

OR – odds ratio, CI – confidence interval

Note: Non-short birth interval was considered the reference category.

*Summary estimates were based on random-effects methods except for the early neonatal mortality and post-neonatal mortality which were based on the fixed-effects model.

†The trim-and-fill method simulates studies that are likely to be missing from the literature due to publication or other forms of bias. The trim-and-fill OR estimates what the pooled OR would be if these missing studies were included in the analysis.

We found evidence of moderate to higher heterogeneity for the likelihoods of stillbirths, perinatal mortality, neonatal mortality, infant mortality, child mortality and U5M (**Table 2**). Stratified analysis was conducted to explore the source of heterogeneity across included characteristics, including sample size, confounding factors, study design and study setting. Results are presented in **Table 3**. We found different likelihoods of stillbirths, perinatal mortality, neonatal mortality, infant mortality, child mortality and U5M across sample size, confounding factors, study design and study setting.

**Table 3:** Stratified analysis of pooled OR of six forms of child mortality across selected characteristics

Characteristics	Stillbirths			Perinatal mortality			Neonatal mortality			Infant mortality			Child mortality			Under-five mortality		
**Pooled OR (95%, CI)**	**Heterogeneity, *P value***	**Meta-regression, *P value***	**Pooled OR (95%, CI)**	**Heterogeneity, *P value***	**Meta-regression, *P value***	**Pooled OR (95%, CI)**	**Heterogeneity, *P value***	**Meta-regression, *P value***	**Pooled OR (95%, CI)**	**Heterogeneity, *P value***	**Meta-regression, *P value***	**Pooled OR (95%, CI)**	**Heterogeneity, *P value***	**Meta-regression, *P value***	**Pooled OR (95%, CI)**	**Heterogeneity, *P value***	**Meta-regression, *P value***
**Sample size**																		
≤21 816	2.20 (1.13-4.29)	<0.05	0.845	1.84 (1.02-3.34)	<0.05	0.781	1.69 (1.40-2.04)	<0.01	<0.05	1.74 (1.42-2.14)	0.227	0.253	1.02 (0.42-2.51)	<0.01	0.033	2.02 (1.09-3.75)	<0.01	0.912
>21 816	1.93 (1.20-3.10)*	NA		1.66 (1.15-2.39)	<0.01		1.97 (1.88-2.07)	<0.01		1.95 (1.81-2.11)	<0.05		1.91 (1.49-2.45)	<0.01		1.93 (1.63-2.28)	<0.01	
**Confounders**																		
Adjusted	2.61 (1.91-3.57)	0.295	<0.01	2.58 (1.61-4.13)*	NA	0.154	1.74 (1.49-2.03)	<0.01	0.463	2.12 (1.57-2.88)	0.131	0.636	1.42 (1.15-1.75)*	NA	0.686	1.80 (1.65-1.97)	0.474	0.830
Unadjusted	1.27 (0.89-1.82)*	NA		1.58 (1.21-2.06)	<0.01		1.90 (1.66-2.17)	<0.01		1.90 (1.75-2.07)	<0.05		1.72 (1.24-2.38)	<0.01		2.01 (1.36-2.97)	<0.01	
**Study design**																		
Prospective cohort	0			2.58 (1.61-4.13)*	NA		1.10 (0.80-1.51)*	NA		0			0			0		
Cross-sectional	1.96 (1.13-3.39)	<0.05	0.522	1.58 (1.21-2.06)	<0.01	0.154	1.90 (1.73-2.08)	<0.01	<0.05	1.90 (1.77-2.05)	<0.05	0.076	1.67 (1.27-2.19)	<0.01	<0.01	1.94 (1.53-2.39)	<0.01	0.869
Case-control	2.99 (1.35-6.62)*	NA		0			0			3.57 (1.82-7.01)*	NA		0			2.08 (1.22-3.57)*	NA	
**Study setting**																		
National	1.93 (1.20-3.10)*	NA	0.845	1.99 (1.68-2.35)*	NA	0.418	1.91 (1.73-2.10)	<0.01	0.082	1.94 (1.82-2.07)	0.118	0.354	1.72 (1.24-2.38)	<0.01	0.686	1.87 (1.46-2.39)	<0.01	0.383
Regional	2.20 (1.13-4.29)	<0.05		1.59 (1.18-2.15)*	<0.01		1.56 (1.10-2.20)	<0.01		2.17 (0.92-5.11)	<0.05		1.42 (1.15-1.75)*	NA		2.46 (1.69-3.59)	<0.01	

OR – odds ratio, CI – confidence interval, NA – not applicable

*Odds ratio for single study.

We also found evidence of publication bias for neonatal mortality (Figure S4a in the **Online Supplementary Document**), infant mortality (Figure S6a in the **Online Supplementary Document**), child mortality (Figure S7a in the **Online Supplementary Document**) and U5M (Figure S8a in the **Online Supplementary Document**). Thus, we conducted the Trim and Fill methods to estimate the number of missing studies. However, after considering the missing studies, the estimated ORs were around similar to the corresponding summary estimates.

Our narrative synthesis also produced a consistent result with the main summary estimates (**Table 4**). Of the ten studies included, six showed a higher prevalence of U5M among babies born after SBIs. Three studies reported SBI as a significant risk factor for child mortality. SBI was also reported as a significant risk factor of infant mortality in two studies.

**Table 4:** Findings from the narrative review of the relationship between short birth interval and six forms of child mortality in low- and lower-middle-income countries, January 2000 to January 2022

Study	Study design, country	Sample	Results
Ejigu AG et al., 2019 [53]	Cross-sectional study, Ethiopia	A total of 418 pregnant women were included during their ANC visits and followed up during pregnancy and after the live births.	The likelihood of child mortality was 3.6 times (aOR = 3.60; 95% CI = 1.35-9.59) higher among babies born in short intervals than babies born in normal intervals.
Hailemichael HT, 2020 [54]	Case-control study, Ethiopia	A total of 405 mothers (135 cases and 270 controls) were recruited from public and private hospitals during delivery care services.	Women who gave birth in SBIs were 5.21 times (aOR = 5.21; 95% CI = 1.89-13.86) more likely to experience child mortality than mothers having normal inter-pregnancy intervals.
Asiki G et al., 2016 [75]	Cohort study, Uganda	Data of a total of 1830 children were analyzed, extracted from census, vital registrations, pregnancy registrations, and medical survey rounds.	Around 55% (HR = 0.45; 95% CI = 3%, 79%) lower risk and 26% (HR = 1.26%-95% CI = 0.40-3.97) higher risk of U5M mortality were found among babies born in 1-2 y and >2 y intervals, respectively, compared to <1 y interval.
Mekonnen Y et al., 2013 [55]	Cross-sectional study, Ethiopia	This study analyzed data of 32 428 children, extracted from the Ethiopian Demography Health Survey.	This study reported 2.2 times (HR = 2.19; 95% CI = 1.89-2.52) likelihood of infant mortality among babies born in SBI than those born in normal intervals.
Kayode GA et al., 2012 [66]	Cross-sectional study, Nigeria	This study analyzed data of 28 647 children, extracted from the Nigerian Demography Health Survey.	Around 51% (OR = 0.49; 95% CI = 0.43-0.56) and 70% (OR = 0.30; 95% CI = 0.26-0.34) lower likelihoods of U5M were found among mothers who gave births in 18-36 mo and >36 mo intervals, respectively, compared to mothers gave birth in <18 mo intervals.
Budu et al., 2021 [17]	Cross- sectional study, eight countries in West Africa	Total of 52 877 childbearing women’s data were analyzed, extracted from nationally representative Demographic and Health Surveys.	The likelihood of under-five mortality was 1.82 (95% CI = 1.64-2.00) times higher among SBI-babies compared to the babies born in normal intervals.
Biradar R et al., 2019 [67]	Cross-sectional study, Nigeria	This study analyzed data of 7468 under-five children, nationally representative Demographic and Health Surveys.	As compared to the mothers having less than 2 y interval in two most recent live births, mothers having 2-3 y and above 3 y interval in their most recent two live births had 6% (aOR = 0.94; 95%CI = 0.79-1.11) and 23% (aOR = 0.77; 95% CI = 0.65-0.92) lower likelihoods of U5M.
Worku MG et al., 2021 [76]	Cross-sectional study, Ethiopia	This study analyzed data of 3446 live births, extracted from nationally representative Demographic and Health Surveys.	The likelihoods of U5M were found 43% (aOR = 0.57; 95%CI = 0.41-0.81) and 65% (aOR = 0.35; 95% CI = 0.22-0.55) lower among mothers having intervals of 2-3 y and above three years in their most recent two live births as compared to the mothers with intervals of less than two years.
Tesma GA et al., 2021 [57]	Cross-sectional study, twelve Est African countries	This study analyzed data of 138 803 under-five children extracted from the Demographic and Health Survey.	The likelihood of U5M (aOR = 0.53; 95%CI = 0.50-0.57) declined among mothers having births intervals of 2-4 y as compared to the mothers having birth intervals of less than two years.
Woldeamanuel BT et al., 2019 [77]	Cross-sectional study, Ethiopia	This study analyzed data of 10 274 under-five children extracted from the Demographic and Health Survey.	Compared to the mothers having birth intervals of 36 mo and more, the likelihoods of U5M were found higher among mothers having birth intervals of 1-18 mo (OR = 2.16; 95% CI = 1.82-2.57) and 19-36 mo (OR = 1.32; 95% CI = 1.18-1.47).

ANC – antenatal care, aOR – adjusted odds ratio, HR – hazard ratio, CI – confidence interval, U5M – under 5 mortality, OR – odds ratio, mo -months

## DISCUSSION

This study aimed to identify the relationship between SBI and several forms of child mortality in LMICs. The results suggest that the likelihoods of various forms of child mortality were 1.58-3.01 times higher among mothers who gave births in short intervals than their counterparts who gave births in normal intervals. These summary effects were found different across study characteristics, including study design, sample size, confounder adjustment and study settings, however, their directions were similar to the corresponding summary estimates. Evidence of publication bias was observed for neonatal mortality, infant mortality, child mortality and U5M, although their corresponding likelihoods were similar before and after adjusting the hypothetical number of studies due to potential publication bias. Overall, our findings show evidence of the adverse effects of SBI on child mortality in LMICs and highlight the critical importance of appropriate policies and programmes to reduce the prevalence of SBI and its adverse effects on child health thereby.

The duration from perinatal mortality to U5M, the eight forms of mortality we examined in this study, covers a period from conception up to 5 years of birth. Our overall findings suggest that across these various bands of durations, the risks of child mortality are around 2-folds in the context of LMICs, except for post-neonatal mortality, the risk for which was 3-folds but estimated from a single study. A recent and large study with data from 77 countries found that birth intervals are an important factor for perinatal outcomes in low-income countries but are much less consequential in high-income settings [78]. In conjunction with the results of this and other similar studies, our findings suggest that a substantial proportion of these deaths attributable to SBIs in LMICs are avoidable. The literature consistently suggests that women’s education, awareness of the adverse effects of SBI on fetuses, babies and mothers and regular antenatal and postnatal care can substantially reduce both SBIs and adverse health outcomes for children and mothers [79,80]. Indeed, the difference between low- and high-income countries in terms of the effects of SBIs on various forms of child mortality is determined by these social, behavioral and public health aspects, which are avoidable.

The underlying reasons for relatively high child mortality among babies born in SBIs touch several aspects, including maternal reproductive health conditions, mother-child behavior, and mothers’ socio-demographic attributes [19,81]. Following a live birth, women need time to return to the normal stage from maternal nutritional depletion and may compromise the ability to support fetal growth, which could result in fetal malnutrition and increased risk of infection and death during childhood. Also, SBI can cause cervical insufficiency, incomplete healing of uterine scars, abnormal remodelling of endometrial blood vessels, ruptured membrane, and anemia [10,12,22]. All these may ultimately contribute to the adverse outcomes in the subsequent pregnancy. Other issues such as competition between short-spaced siblings for parental time, material resources and inadequate parental attention have also been identified as possible causes of child mortality among babies born in SBIs [6,82]. These socio-behavioral issues make infants susceptible to infectious diseases (eg, diarrhea, acute respiratory infection) and child nutritional disorders (stunting, wasting, and underweight) [83-85], which, in LMICs, are major causes of child mortality [86].

Another leading cause of the relatively high prevalence of SBI in LMICs is unintended conceptions. The prevalence of unintended pregnancies is higher among the disadvantaged women than their advantaged counterparts, mainly due to infrequent use of modern contraception [87], ineffective contraceptives use [88], inadequate use of emergency contraception [89], or combinations of these three. Many of these women suffer from depression due to unintended pregnancies, and they have limited access to antenatal health care, delivery health care and postnatal health care. Moreover, disadvantaged women, in general, and women having SBI, in particular, tend to depend on their prior pregnancy experiences [90]. In addition, they are more likely to face cultural restrictions in utilizing maternal health care services and have usually lower knowledge about the adverse child health outcomes [5]. Negative effects of unintended conception are continued following the live birth and affect preventative and curative care of the newborn, including exclusive breastfeeding and newborn care [91]. The adverse effects may exacerbate further since mothers giving birth in SBIs may not be able to ensure adequate care for two infants of almost the same age; and ultimately, all these, together, can increase the risk of child mortality.

A previous systematic review conducted based on the studies published in the Ethiopian context reported a higher likelihood of infant mortality among babies born in SBIs (OR = 2.03; 95% CI = 1.52-2.70) [24] than the estimate we found (OR = 1.88; 95% CI = 1.71, 2.08). This difference could be due to the sample variation since that study summarized papers only in the Ethiopian context, where a higher parity and lower use of maternal health care services are more prevalent than many other LMICs [15,16]. Since our summary estimates for the effects of SBI on U5M, neonatal mortality and stillbirths are the first in the context of LMICs, we are unable to compare them.

This study has several strengths and some limitations. As far we know, this is the first study in LMICs that summarized the effects of SBI on various forms of child mortality. All studies conducted in LMICs since the year 2000, when the MDGs were established aiming to reduce maternal and child mortality, were included in this review. Moreover, we used comprehensive search techniques for data extraction and selection of eligible studies and followed the “Strengthening the Reporting of Observational Studies in Epidemiology” and PRISMA guidelines for reporting study findings. In addition, we considered all forms of child mortality and analyzed them separately following the relevant international classifications. However, the summary estimates presented in this study were mainly based on cross-sectional studies. We did not search unpublished papers and gray literature, some of which could contribute to publication bias, although it is likely to be insignificant as Egger’s test and funnel plots suggest. The slight asymmetry that we observed may arise due to the inclusion of a small number of studies on particular forms of mortality, substantial between-study heterogeneity and/or similar studies size. However, we found a similar trend in results of summarized ORs when we captured publication bias by using the trim-and-fill method, thus suggesting that our findings are reliable.

## CONCLUSIONS

This study found significantly higher likelihoods of several forms of child mortality among babies born in short intervals than their counterparts born in normal intervals. These indicate SBI is an important public health challenge in LMICs for achieving a significant reduction in child mortality in the current round of the world development goals, the SDGs, to be achieved by 2030. Policies and programs need to be strengthened to improve reproductive and maternal health care services, reduce the occurrences of short interval births and ensure health care services in every pregnancy. Initiatives are also needed to increase awareness about the adverse effect of short interval birth.

## Additional material


Online Supplementary Document
