# Supplementary material for: Negative effects of short birth interval on child mortality in low- and middle-income countries: A systematic review and meta-analysis
Source: J Glob Health. 2022 Sep 3;12:04070. doi: 10.7189/jogh.12.04070 (PMC9441110; doi:10.7189/jogh.12.04070)
Supplement: Online Supplementary Document [file jogh-12-04070-s001.pdf]

Online supplemental material

Effects of Short Birth Interval on Child Mortality in Low- and Middle Income Countries: A systematic review and meta-analysis

Table S1. Search strategy of Medline database (date: January 2000 to January 2022)

#	Searches	Results
1	Birth Interval*.mp. or Short birth interval.mp. or pregnancy interval.mp. or inter-pregnancy interval.mp.	1821
2	stillbirths.mp. or Stillbirth/	9371
3	neonatal mortality.mp. or early-neonatal mortality.mp. or post-neonatal mortality.mp. or Infant Mortality/	33362
4	perinatal mortality.mp. or Perinatal Mortality/	10868
5	under-five mortality.mp. or under five mortality.mp. or U5M mortality.mp.	15023
6	2 or 3 or 4 or 5	62023
7	(Afghani* or Guinea* or Rwand* or Benin* or Guinea-Bissau* or Senegal* or Burkina Faso* or Haiti* or Sierra Leone* or Burundi* or Korea* or Somalia* or Central African Republic or Liberia* or South Sudan* or Chad* or Madagasca* or Tanzania* or Comoros* or Malawi* or Togo* or Congo* or Mali* or Uganda* or Eritrea* or Mozambique* or Zimbabwe* or Ethiopia* or Nepa* or Gambia* or Niger* or Angol* or Indonesia* or Philippin* or Armenia* or Jordan* or Sao Tome* or Bangladesh* or Kenya* or Solomon Island* or Bhutan* or Kiribati* or Sri Lanka* or Bolivia* or Kosov* or Sudan* or Cabo Verde* or Kyrgyz Republic* or Swazi* or Cambodia* or Lao* or Syria* or Cameroon* or Lesotho* or Tajikistan* or Congo* or Mauritania* or Timor-Leste* or Cote d'Ivoire* or Micronesia* or Tunisia* or Djibouti* or Moldov* or Ukrain* or Egypt* or Mongolia* or Uzbekistan* or El Salvador* or Morocc* or Vanuat* or Georgia* or Myanmar* or Vietnam* or Ghana* or Nicaragua* or West Bank* or Gaza* or Guatemala* or Nigeria* or Yemen* or Hondur* or Pakistan* or Zambia* or India* or Papua New Guinea*).mp. [mp=title, abstract, original title, name of substance word, subject heading word, floating sub-heading word, keyword heading word, organism supplementary concept word, protocol supplementary concept word, rare disease supplementary concept word, unique identifier, synonyms]	1619699
8	1 and 6 and 7	1169
9	limit 25 to yr="2000 -Current"	921

Table S2. Search strategy of Embase database (date: January 2000 to January 2022)

#	Searches	Results
1	Birth Interval*.mp. or Short birth interval.mp. or pregnancy interval.mp. or inter-pregnancy interval.mp.	41009
2	stillbirths.mp. or Stillbirth/	23225
3	neonatal mortality.mp. or Infant Mortality/	34393
4	perinatal mortality.mp. or Perinatal Mortality/	20567
5	under-five mortality.mp. or under five mortality.mp. or U5M mortality.mp.	21035
6	2 or 3 or 4 or 5	82056
7	(Afghani* or Guinea* or Rwand* or Benin* or Guinea-Bissau* or Senegal* or Burkina Faso* or Haiti* or Sierra Leone* or Burundi* or Korea* or Somalia* or Central African Republic or Liberia* or South Sudan* or Chad* or Madagasca* or Tanzania* or Comoros* or Malawi* or Togo* or Congo* or Mali* or Uganda* or Eritrea* or Mozambique* or Zimbabwe* or Ethiopia* or Nepa* or Gambia* or Niger* or Angol* or Indonesia* or Philippin* or Armenia* or Jordan* or Sao Tome* or Bangladesh* or Kenya* or Solomon Island* or Bhutan* or Kiribati* or Sri Lanka* or Bolivia* or Kosov* or Sudan* or Cabo Verde* or Kyrgyz Republic* or Swazi* or Cambodia* or Lao* or Syria* or Cameroon* or Lesotho* or Tajikistan* or Congo* or Mauritania* or Timor-Leste* or Cote d'Ivoire* or Micronesia* or Tunisia* or Djibouti* or Moldov* or Ukrain* or Egypt* or Mongolia* or Uzbekistan* or El Salvador* or Morocc* or Vanuat* or Georgia* or Myanmar* or Vietnam* or Ghana* or Nicaragua* or West Bank* or Gaza* or Guatemala* or Nigeria* or Yemen* or Hondur* or Pakistan* or Zambia* or India* or Papua New Guinea*).mp. [mp=title, abstract, heading word, drug trade name, original title, device manufacturer, drug manufacturer, device trade name, keyword, floating subheading word, candidate term word]	2367427
8	1 and 6 and 7	487

9	limit 25 to yr="2000 -Current"	389

Table S3. Search strategy of APA PsychINFO database (date: January 2000 to January 2022)

#	Searches	Results
1	Birth Intervals/ or Short birth interval.mp.	7
2	stillbirths.mp. or Stillbirth/	245
3	neonatal mortality.mp. or Infant Mortality/	339
4	perinatal mortality.mp. or Perinatal Mortality/	217
5	under-five mortality.mp. or under five mortality.mp. or U5M mortality.mp.	201
6	2 or 3 or 4 or 5	856
7	(Afghani* or Guinea* or Rwand* or Benin* or Guinea-Bissau* or Senegal* or Burkina Faso* or Haiti* or Sierra Leone* or Burundi* or Korea* or Somalia* or Central African Republic or Liberia* or South Sudan* or Chad* or Madagasca* or Tanzania* or Comoros* or Malawi* or Togo* or Congo* or Mali* or Uganda* or Eritrea* or Mozambique* or Zimbabwe* or Ethiopia* or Nepa* or Gambia* or Niger* or Angol* or Indonesia* or Philippin* or Armenia* or Jordan* or Sao Tome* or Bangladesh* or Kenya* or Solomon Island* or Bhutan* or Kiribati* or Sri Lanka* or Bolivia* or Kosov* or Sudan* or Cabo Verde* or Kyrgyz Republic* or Swazi* or Cambodia* or Lao* or Syria* or Cameroon* or Lesotho* or Tajikistan* or Congo* or Mauritania* or Timor-Leste* or Cote d'Ivoire* or Micronesia* or Tunisia* or Djibouti* or Moldov* or Ukrain* or Egypt* or Mongolia* or Uzbekistan* or El Salvador* or Morocc* or Vanuat* or Georgia* or Myanmar* or Vietnam* or Ghana* or Nicaragua* or West Bank* or Gaza* or Guatemala* or Nigeria* or Yemen* or Hondur* or Pakistan* or Zambia* or India* or Papua New Guinea*).mp. [mp=title, abstract, heading word, table of contents, key concepts, original title, tests & measures, mesh]	16070
8	1 and 6 and 7	1003
9	limit 25 to yr="2000 -Current"	192

Table S4. Search strategy in Maternity and Infant care database (date: January 2000 to January 2022)

Results	Type	Actions
1	[Birth Intervals/ or Short birth interval.mp.]	112
2	[stillbirths.mp. or Stillbirth/]	17
3	[neonatal mortality.mp. or Infant Mortality/]	22
4	[perinatal mortality.mp. or Perinatal Mortality/]	95
5	[under-five mortality.mp. or under five mortality.mp. or U5M mortality.mp.]	78
6	2 or 3 or 4 or 5	80
7	(Afghani* or Guinea* or Rwand* or Benin* or Guinea-Bissau* or Senegal* or Burkina Faso* or Haiti* or Sierra Leone* or Burundi* or Korea* or Somalia* or Central African Republic or Liberia* or South Sudan* or Chad* or Madagasca* or Tanzania* or Comoros* or Malawi* or Togo* or Congo* or Mali* or Uganda* or Eritrea* or Mozambique* or Zimbabwe* or Ethiopia* or Nepa* or Gambia* or Niger* or Angol* or Indonesia* or Philippin* or Armenia* or Jordan* or Sao Tome* or Bangladesh* or Kenya* or Solomon Island* or Bhutan* or Kiribati* or Sri Lanka* or Bolivia* or Kosov* or Sudan* or Cabo Verde* or Kyrgyz Republic* or Swazi* or Cambodia* or Lao* or Syria* or Cameroon* or Lesotho* or Tajikistan* or Congo* or Mauritania* or Timor-Leste* or Cote d'Ivoire* or Micronesia* or Tunisia* or Djibouti* or Moldov* or Ukrain* or Egypt* or Mongolia* or Uzbekistan* or El Salvador* or Morocc* or Vanuat* or Georgia* or Myanmar* or Vietnam* or Ghana* or Nicaragua* or West Bank* or Gaza* or Guatemala* or Nigeria* or Yemen* or Hondur* or Pakistan* or Zambia* or India* or Papua New Guinea*).mp. [mp=abstract, heading word, title]	1786
8	1 and 6 and 7	62
9	limit 25 to yr="2000 -Current"	44

Table S5. Search strategy of web of science (WOS) database (date: January 2000 to January 2022)

1	Birth Intervals/ or Short birth interval.mp.	39469
2	stillbirths.mp. or Stillbirth/	21069
3	neonatal mortality.mp. or Infant Mortality/	32683
4	perinatal mortality.mp. or Perinatal Mortality/	19853
5	under-five mortality.mp. or under five mortality.mp. or U5M mortality.mp.	21053
6	2 or 3 or 4 or 5	32023
7	(Afghani* or Guinea* or Rwand* or Benin* or Guinea-Bissau* or Senegal* or Burkina Faso* or Haiti* or Sierra Leone* or Burundi* or Korea* or Somalia* or Central African Republic or Liberia* or South Sudan* or Chad* or Madagasca* or Tanzania* or Comoros* or Malawi* or Togo* or Congo* or Mali* or Uganda* or Eritrea* or Mozambique* or Zimbabwe* or Ethiopia* or Nepa* or Gambia* or Niger* or Angol* or Indonesia* or Philippin* or Armenia* or Jordan* or Sao Tome* or Bangladesh* or Kenya* or Solomon Island* or Bhutan* or Kiribati* or Sri Lanka* or Bolivia* or Kosov* or Sudan* or Cabo Verde* or Kyrgyz Republic* or Swazi* or Cambodia* or Lao* or Syria* or Cameroon* or Lesotho* or Tajikistan* or Congo* or Mauritania* or Timor-Leste* or Cote d'Ivoire* or Micronesia* or Tunisia* or Djibouti* or Moldov* or Ukrain* or Egypt* or Mongolia* or Uzbekistan* or El Salvador* or Morocc* or Vanuat* or Georgia* or Myanmar* or Vietnam* or Ghana* or Nicaragua* or West Bank* or Gaza* or Guatemala* or Nigeria* or Yemen* or Hondur* or Pakistan* or Zambia* or India* or Papua New Guinea*).mp. [mp=title, abstract, heading word, drug trade name, original title, device manufacturer, drug manufacturer, device trade name, keyword, floating subheading word, candidate term word]	112820
8	1 and 6 and 7	249
10	limit 25 to yr="2000 -Current"	160

Table S6. Search strategy in CINAHL database **(date: January 2000 to January 2022)**

S1	Birth Intervals or Birth spacing or Short birth interval	Expanders - Apply equivalent subjects Search modes - Boolean/Phrase	1492
S2	stillbirths.mp. or Stillbirth	Expanders - Apply equivalent subjects Search modes - Boolean/Phrase	2512
S3	neonatal mortality or Infant Mortality	Expanders - Apply equivalent subjects Search modes - Boolean/Phrase	1402
S4	perinatal mortality or Perinatal Mortality	Expanders - Apply equivalent subjects Search modes - Boolean/Phrase	1702
S5	under-five mortality or under five mortality or U5M mortality	Expanders - Apply equivalent subjects Search modes - Boolean/Phrase	1208
S6	2 or 3 or 4 or 5	Expanders - Apply equivalent subjects Search modes - Boolean/Phrase	1982
S7	(Afghani* or Guinea* or Rwand* or Benin* or Guinea-Bissau* or Senegal* or Burkina Faso* or Haiti* or Sierra Leone* or Burundi* or Korea* or Somalia* or Central African Republic or Liberia* or South Sudan* or Chad* or Madagasca* or Tanzania* or Comoros* or Malawi* or Togo* or Congo* or Mali* or Uganda* or Eritrea* or Mozambique* or Zimbabwe* or Ethiopia* or Nepa* or Gambia* or Niger* or Angol* or Indonesia* or Philippin* or Armenia* or Jordan* or Sao Tome* or Bangladesh* or Kenya* or Solomon Island* or Bhutan* or Kiribati* or Sri Lanka* or Bolivia* or Kosov* or Sudan* or Cabo Verde* or Kyrgyz Republic* or Swazi* or Cambodia* or Lao* or Syria* or Cameroon* or Lesotho* or Tajikistan* or Congo* or Mauritania* or Timor-Leste* or Cote d'Ivoire* or Micronesia* or Tunisia* or Djibouti* or Moldov* or Ukrain* or Egypt* or Mongolia* or Uzbekistan* or El Salvador* or Morocc* or Vanuat* or Georgia* or Myanmar* or Vietnam* or Ghana* or Nicaragua* or West Bank* or Gaza* or Guatemala* or Nigeria* or Yemen* or	Expanders - Apply equivalent subjects Search modes - Boolean/Phrase	2570253

	Hondur* or Pakistan* or Zambia* or India* or Papua New Guinea*).		
S8	S1 AND S6 AND S7		160
S9	S8	Limiters - Published Date: >20000101<br><br>Expanders - Apply equivalent subjects<br><br>Search modes - Boolean/Phrase	102

Table S7. Search strategy in the Cochrane Library database (**date: January 2000 to January 2022**)

Searches	Results
Birth Intervals or Birth spacing or Short birth interval in Title Abstract Keyword AND (stillbirth* or neonatal mortality or perinatal mortality or under-five mortality or U5M in Title Abstract Keyword) AND (Afghani* or Guinea* or Rwand* or Benin* or Guinea-Bissau* or Senegal* or Burkina Faso* or Haiti* or Sierra Leone* or Burundi* or Korea* or Somalia* or Central African Republic or Liberia* or South Sudan* or Chad* or Madagasca* or Tanzania* or Comoros* or Malawi* or Togo* or Congo* or Mali* or Uganda* or Eritrea* or Mozambique* or Zimbabwe* or Ethiopia* or Nepa* or Gambia* or Niger* or Angol* or Indonesia* or Philippin* or Armenia* or Jordan* or Sao Tome* or Bangladesh* or Kenya* or Solomon Island* or Bhutan* or Kiribati* or Sri Lanka* or Bolivia* or Kosov* or Sudan* or Cabo Verde* or Kyrgyz Republic* or Swazi* or Cambodia* or Lao* or Syria* or Cameroon* or Lesotho* or Tajikistan* or Congo* or Mauritania* or Timor-Leste* or Cote d'Ivoire* or Micronesia* or Tunisia* or Djibouti* or Moldov* or Ukrain* or Egypt* or Mongolia* or Uzbekistan* or El Salvador* or Morocc* or Vanuat* or Georgia* or Myanmar* or Vietnam* or Ghana* or Nicaragua* or West Bank* or Gaza* or Guatemala* or Nigeria* or Yemen* or Hondur* or Pakistan* or Zambia* or India* or Papua New Guinea*). in Title Abstract Keyword - (Word variations have been searched) AND Limiters - Published Date: >20000101	2

Table S8. Search strategy of Scopus database (**date: January 2000 to January 2022**)

Searches	Result
((TITLE-ABS-KEY (Birth AND intervals) OR TITLE-ABS-KEY (Birth AND spacing) OR TITLE-ABS-KEY ((short OR birth OR interval)) AND ((stillbirths OR (early AND neonatal AND mortality) OR (post AND neonatal AND mortality) OR (neonatal AND mortality) OR (perinatal AND mortality) OR (infant AND mortality) OR (under-five AND mortality) OR (U5M) OR (under five AND mortality)) AND ((TITLE-ABS-KEY (afghani* OR guinea* OR rwand* OR benin* OR guinea-bissau* OR senegal* OR burkina AND faso* OR haiti* OR sierra AND leone* OR burundi* OR korea* OR somalia* OR central AND african AND republic OR liberia* OR south AND sudan* OR chad* OR madagasca* OR tanzania* OR comoros*) OR TITLE-ABS-KEY (malawi* OR togo* OR congo* OR mali* OR uganda* OR eritrea* OR mozambique* OR zimbabwe* OR ethiopia* OR nepa* OR gambia* OR niger*) OR (angol* OR indonesia* OR philippin* OR armenia* OR jordan* OR sao AND tome* OR bangladesh* OR kenya* OR solomon AND island* OR bhutan* OR kiribati* OR sri AND lanka* OR bolivia* OR kosov* OR sudan* OR cabo AND verde* OR kyrgyz AND republic* OR swazi* OR cambodia* OR lao*) OR TITLE-ABS-KEY (syria* OR cameroon* OR lesotho* OR tajikistan* OR congo* OR mauritania* OR timor leste* OR cote AND d'ivoire* OR micronesia* OR tunisia* OR djibouti* OR moldov* OR ukrain* OR egypt* OR mongolia* OR uzbekistan* OR el AND salvador* OR morocc*) OR TITLE-ABS-	136

KEY (vanuat* OR georgia* OR myanmar* OR vietnam* OR ghana* OR nicaragua* OR west AND bank* OR gaza* OR guatemala* OR nigeria* OR yemen* OR hondur* OR pakistan* OR zambia* OR india* OR pap ua AND new AND guinea*)) AND PUBYEAR > 2000)	

Supplemental Table S9. Summary of observational studies on short birth interval and several form of child mortality covering studies conducted in low- and lower-middle income countries, January 2000 to January, 2022.

**Authors, year, and country**	**Study design and settings**	**Sample**	**Outcomes**	**Confounders adjustment**
Morakinyo OM & Fagbamigbe AF, 2017 (1), Nigeria	Cross sectional study, National	66,158	Neonatal mortality, Infant mortality, Under-five mortality	Age, marital status, wealth status, education, place of residence, sex of the child, birth order, ANC visits, skilled birth attendants
Dagne HM et al., 2021 (2), Ethiopia	Case control study, Institutional	402	Stillbirths	Time of first ANC visit, hypertensive disorder of pregnancy, partograph used, duration of labour, mode of delivery, birth weight
Akinyemi JO et al., 2015 (3), Nigeria	Cross sectional study, National	57340	Neonatal mortality	Region, urban residence, maternal education, marital status, improved drinking water source, birth order
Akter S et al., 2010 (4), Bangladesh	Cross sectional study, National	8090	Infant mortality, Child mortality	Place of residence, education, religion, occupation, age, age at birth, breastfeeding, wealth index, birth order
Lakew D et al., 2017 (5), Ethiopia	Cross sectional study, Regional	2555	Stillbirths	Residence, age, education, wealth index, ANC visits, Number of ANC visits, Use modern contraceptive, type of contraceptives, place of delivery
Fenta SM et al, 2021 (6), Ethiopia	Cross sectional study, National	2449	Neonatal mortality	Sex of neonate, age of respondents at first birth, mothers' education, fathers' education, wealth index, residence, region, number of ANC visits, Pregnancy duration, birth order, TT injections, type of birth
Lamichhane R et al., 2017 (7), Nepal	Cross sectional study, National	11,110	Infant mortality	Region, ecological zone, socioeconomic factors, maternal education, paternal education, wealth Index, sex of child, birth order
Andargie et al, 2013 (8), Ethiopia	Prospective cohort study, Institutional	1752	Perinatal mortality	Marital status, education of mother and father, mother's occupation, father's occupation, sex of infants, type of birth outcome, history of abortion, previous still births

Jonge HCCD et al., 2014 (9), Bangladesh	Cross sectional study, Regional	5571	Perinatal mortality, Stillbirths, Neonatal mortality, Early neonatal mortality	Age at onset of birth interval, parity at onset of birth interval, age at first pregnancy, religion, education, household assets
Ahmed Z et al., 2016 (10), Pakistan	Cross sectional study, National	23581	Child mortality	Region, mother's education, breastfeeding duration
Ezeh OK et al., 2019 (11), Nigeria	Cross sectional study, National	24607	Perinatal mortality	Mother's age, mother's body mass index, residence type, sex of index child, baby size at birth, birth rank
Houweling TAJ et al, 2018 (12), Bangladesh and India	Cross sectional study, Regional	6213	Neonatal mortality	Unadjusted
Fotso JC et al, 2013 (13), Kenya	Cross sectional study, Regional	13502	Infant mortality, Child mortality	Mother's age, mother's education, wealth status, sex of index child
Starnes JR et al, 2018 (14), Kenya	Cross sectional study, Regional	428	Under-five mortality	Birth year, maternal age, marital status, Has cell phone, Improved pit latrine, Have livestock, Born in rainy season, multiple gestation pregnancy
Tesema GA et al, 2021 (15), Ethiopia	Cross sectional study, National	11022	Infant mortality	Residence, sex of household head, wealth index, parity, maternal BMI, Health insurance, sex of child, place of delivery, maternal age, education
Tessema ZT et al, 2020 (16), Ethiopia	Cross sectional study, National	11022	Neonatal mortality	Residence, maternal age, education, wealth status, sex of household head, type of birth, parity, size of neonate birth, media exposure, health insurance, and TT vaccine.
Hossain MM et al., 2015 (17), Bangladesh	Cross sectional study, National	24608	Child mortality	Age, place of residence, education, socioeconomic status, children ever born, and mode of delivery
Patel KK et al, 2021 (18), Nigeria	Cross sectional study, National	27465	Neonatal mortality, Infant mortality	Institutional delivery, sex of child, birth size, wealth index, and religion
Khan MA et al, 2021 (19), Bangladesh	Cross sectional study, National	7886	Under-five mortality	Maternal current age, maternal education, residence, economic status, employment status, birth order, ANC, and PNC
Adedini SA et al, 2015 (20), Nigeria	Cross sectional study, National	28647	Infant mortality, Child mortality	Maternal age, maternal education, region of residence, place of residence, child's sex, birth

				order, wealth index and religion
Basha GW et al, 2020 (21), Ethiopia	Cross sectional study, National	11023	Neonatal mortality	Maternal age, maternal education, paternal education, wealth index, place of residence, sex of child, birth order, birth type, birth size, religion, and ANC.
Afshan K et al., 2019 (22), Pakistan	Cross sectional study, National		Neonatal mortality, Post neonatal mortality, Infant mortality, Child mortality, Under-five mortality	Age of mother, mothers' education, place of residence, region, wealth quintile, birth order, birth size, and sex of child.
Kidus F et al, 2019 (23), Ethiopia	Cross sectional study, Institutional	238	Neonatal mortality	Age of mother, educational status, religion, occupation, family size, monthly income, and ethnicity.
Ahmed Z et al., 2016(24), Pakistan	Cross sectional study, National		Child mortality	Region, education of mother, birth order, size of child at birth, breastfeeding, and family size.
Kayode GA et al., 2014 (25), Ghana	Cross sectional study, National	5303	Neonatal mortality	Maternal age, maternal education, maternal occupation, paternal education, paternal occupation, wealth index, birth order, birth weight, breastfeeding and infant sex.
Sahu D et al, 2015 (26), India	Cross sectional study, National	11177	Infant mortality, Child mortality	Mothers' age, mothers' education, mothers' work status, region, sex of child, birth order, and ANC.
Tesema GA et al, 2020 (27), Ethiopia	Cross sectional study, National	11375	Stillbirth	Residence, region, religion, wealth status, education, birth order, and ANC.
Mahande MJ & Obure J, 2016 (28), Tanzania	Cross sectional study, Institutional	20343	Perinatal mortality	Preterm birth and low birth weight.
Tesfye B et al, 2017 (29), Ethiopia	Cross sectional study, National	11654	Child mortality	Age of mother, age of mother at first birth, maternal education, occupation of mother, and place of residence.
Ezeh OK et al., 2014 (30) Nigeria	Cross sectional study, National	27147	Neonatal mortality	Mother's age, mother's age at first birth, mother's education, mother's working status, mother's body mass index, residence, religion, and household wealth index.
Nisar YB & Dibley MJ, 2014	Cross sectional study,	5702	Neonatal mortality	Maternal age, region, parental education, parental

(31), Pakistan	National			occupation, household wealth index, and child's sex.
Shifa GT et al, 2018 (32), Ethiopia	Case control study, Regional	1149	Infant mortality, Under-five mortality	Age of mother, birth order, history of child death before index child, history of child birth after index child, and type of birth.
Tesema GA & Worku MG, 2021 (33), Ethiopia	Cross sectional study, National	4238	Neonatal mortality	Sex of neonate, wealth index, birth order, maternal education, residence, distance to health facility, and ANC.
Haq I et al, 2020 (34), Bangladesh	Cross sectional study, Rural	51791	Child mortality	Women's age, age at first marriage, women's education, husband age, wealth status, religion, division, and children ever born.
Amir-ud-Din R et al., 2022 (35), Pakistan	Cross sectional study, National	12769	Under-five mortality	Mothers' age, place of residence, respondent's education, husband's education, size of child at birth, sex of child, children ever born, and wealth quintile.
Fagbamagbe AF & Nnanutu CC, 2021(36), Nigeria	Cross sectional study, National	33924	Under-five mortality	Mothers' age, mothers' education, child's sex, wealth quintile, birth order, mothers' employment, and accessing health care.
Shifti DM et al., 2021 (37), Ethiopia	Cross sectional study, National	8448	Neonatal mortality, Infant mortality, Under-five mortality	Mothers' age, mothers' education, mothers' occupation, husbands' education, husbands' occupation, wealth quintile, residence, and region.
Yaya S et al., 2020 (38), Benin	Cross sectional study, National	5977	Under-five mortality	Mother's education, mother's occupation, husband's education, husband's occupation, sex of child, wealth quintile and place of residence and region.
Kibria GM et al., 2018 (39), Bangladesh	Prospective cohort study, Regional	21227	Neonatal mortality	Sex of child, birth order, maternal age, history of child death, antenatal care, maternal education, paternal education, and wealth quintile.
Kibria GM et al., 2018 (40), Afganistan	Cross sectional study, National	19801	Neonatal mortality	Maternal age, sex of the child, birth order, wealth quintile, maternal education, paternal education, place of residence, and region.
Yaya S et al., 2020 (41), 34	Cross sectional study,	299065	Infant mortality	Unadjusted

Sub-Saharan African countries	National			

Quality assessment of the included studies

Supplemental Table S10. Newcastle-Ottawa scale assessment of study quality for **cross-sectional study**

Author	Selection					Comparability		Outcome		Study quality
	1	2	3	4		5		6	7	
	Representativeness of the sample	Sample size	Ascertainment of exposure	Non-respondents		The subjects in different outcome groups are comparable, based on the study design or analysis. Confounding factors are controlled.		Assessment of outcome	Statistical test is appropriate	
Morakinyo and Fagbamigbe, 2017, Nigeria	*	*	*	*		*		*	*	7
Akinyemi et al., 2015, Nigeria	*	*	*	*		*		*	*	7
Akter et al., 2010, Bangladesh	*	*	*	*		*		*	*	7
Lakew et al., 2017, Ethiopia	*	*		*		*		*	*	6
Fenta et al., 2021, Ethiopia	*	*	*	*		*		*	*	7
Lamichhane et al.,	*	*		*		*		*	*	6

2017, Nepal										
Jonge et al., 2014, Bangladesh	*	*	*	*		*		*	*	7
Ahmed et al., 2016, Pakistan	*	*	*	*		*		*	*	7
Ezeh et al., 2019, Nigeria	*	*	*	*		*		*	*	7
Houwelling et al., 2018, Bangladesh and India	*	*	*	*				*	*	6
Fotso et al., 2012, Kenya	*	*	*	*		*		*	*	7
Starnes et al., 2012, Kenya	*	*	*	*		*		*	*	7
Tesema et al., 2021, Ethiopia	*	*	*	*		*		*	*	7
Tesema et al., 2020, Ethiopia	*	*	*	*		*		*	*	7
Hossain et al., 2015, Bangladesh	*	*	*	*		*		*	*	7
Patel et al, 2020, Nigeria	*	*	*	*		*		*	*	7
Khan et al., 2021, Bangladesh	*	*	*	*		*		*	*	7

Adedini et al., 2015, Nigeria	*	*	*	*		*		*	*	7
Basha et al., 2020, Ethiopia	*	*	*	*		*		*	*	7
Afshan et al., 2019, Pakistan	*	*	*	*		*		*	*	7
Kidus et al., 2019, Ethiopia			*	*		*		*	*	5
Ahmed et al., 2016, Pakistan	*	*	*	*		*		*	*	7
Kayode et al., 2014, Ghana	*	*	*	*		*		*	*	7
Sahu et al., 2015, India	*	*	*	*		*		*	*	7
Tesema GA et al., 2020, Ethiopia	*	*	*	*		*		*	*	7
Mahande and Obure, 2016, Tanzania	*	*	*	*		*		*	*	7
Tesfye et al., 2017, Ethiopia	*	*	*	*		*		*	*	7
Ezeh OK et al., 2014, Nigeria	*	*	*	*		*		*	*	7
Nasir and Dibley, 2014, Pakistan	*	*	*	*		*		*	*	7

Tesema and Worku, 2021, Ethiopia	*	*	*	*		*		*	*	7
Haq et al., 2020, Bangladesh	*	*	*	*		*			*	6
Amir-ud-Din et al., 2022, Pakistan	*	*	*	*		*			*	6
Fagbamagbe AF & Nnanutu CC, 2021, Nigeria	*	*	*	*		*		*	*	7
Shifti DM et al., 2021, Ethiopia	*	*	*	*		*		*	*	7
Yaya S et al., 2020, Benin	*	*	*	*		*			*	6
Kibria GM et al., 2018, Afganistan	*	*	*	*		*		*	*	7
Yaya S et al., 2020, 34 Sub-Saharan African countries	*	*	*	*				*	*	6

Supplemental Table S11. Newcastle-Ottawa scale assessment of study quality for **case-control study**

Author	Selection					Comparability			Exposure			Stud y quali
	1	2	3	4		5A	5B		6	7	8	

	Is the case definition adequate?	Representativeness of the cases	Selection of controls	Definition of controls		Case-control comparable on basis of age	Case-control comparable on other factor(s)		Ascertainment of exposure	Same method of ascertainment for cases and control	Non-response rate	ty
Dagne et al., 2021, Etiopia			*	*		*	*		*	*	*	7
Shifa et al. 2018, Etiopia	*	*	*	*			*		*	*	*	8

Supplemental Table S12. Newcastle-Ottawa scale assessment of study quality for **cohort study**

Author	Selection					Comparability			Outcome			Study quality
	1	2	3	4		5A	5B		6	7	8	
	Exposed cohort truly representative	Non-exposed cohort drawn from the same community	Ascertain-ment of exposure	Outcome of interest not present at start		Cohorts comparable on basis of age	Cohorts comparable on other factor(s)		Quality of outcome assessment	Follow-up long enough for outcomes to occur	Complete accounting for cohorts	
Andargie et al., 2013		*	*	*			*		*	*	*	7
Kibria GM et al., 2018, Bangladesh	*											

Online supplemental material

Online supplemental material

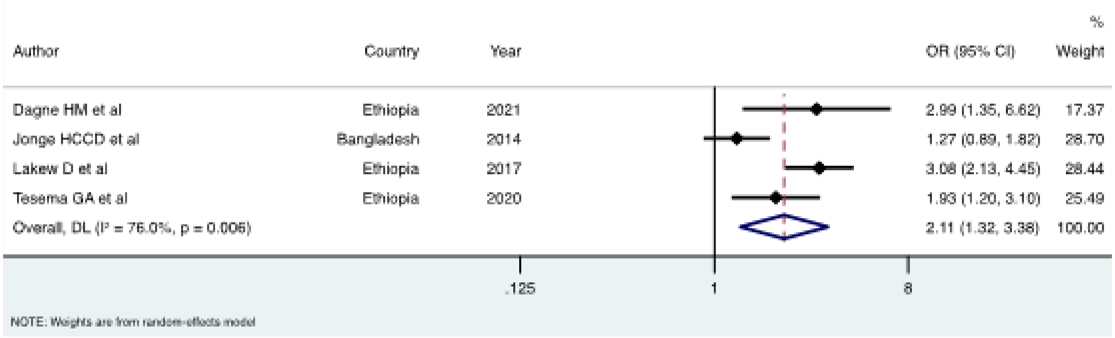

Supplementary Figure S1: Summary effect of short birth interval on stillbirths for low- and lower middle-income countries, January 2000 to January 2022

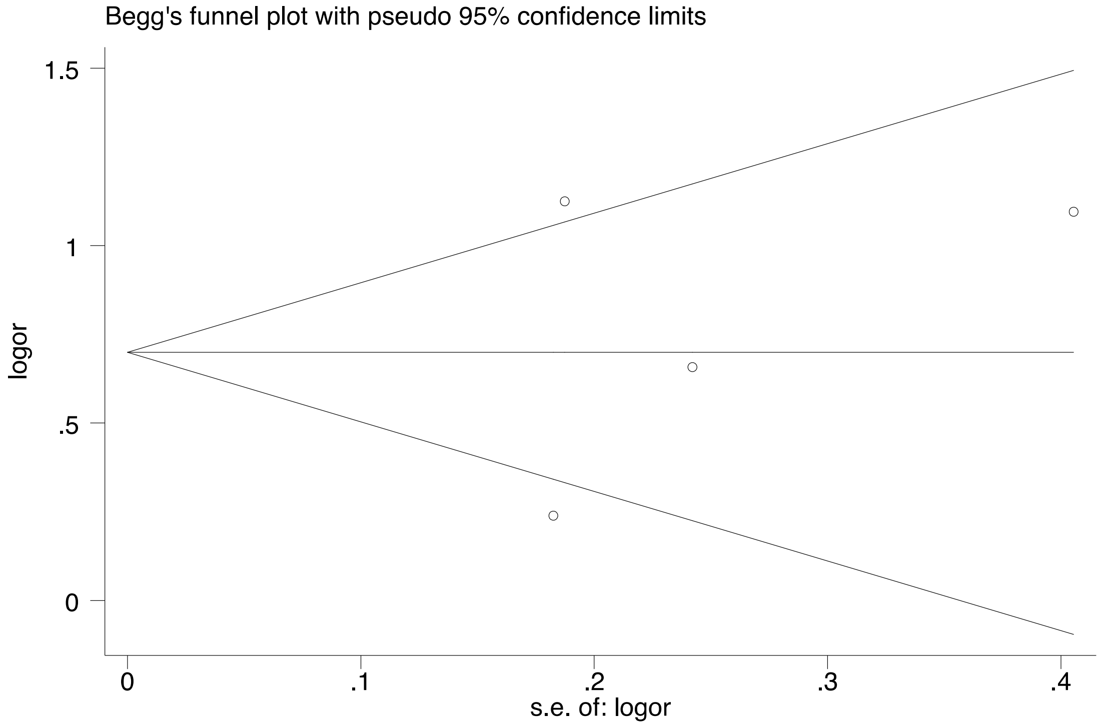

Supplementary Figure S1a: Evidence of publication bias in studies on stillbirth

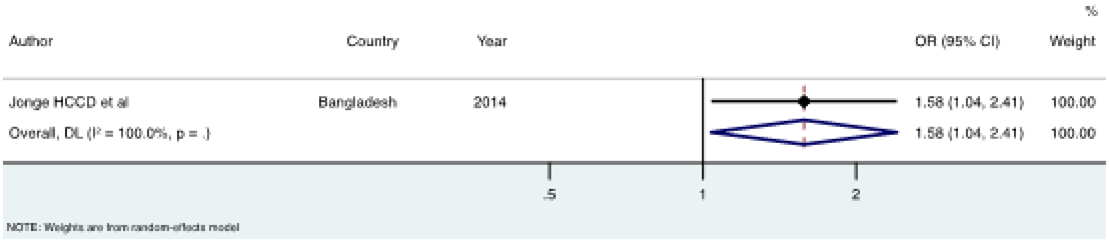

Supplementary Figure S2: Summary effect of short birth interval on early neonatal mortality for low- and lower middle-income countries, January 2000 to January 2022

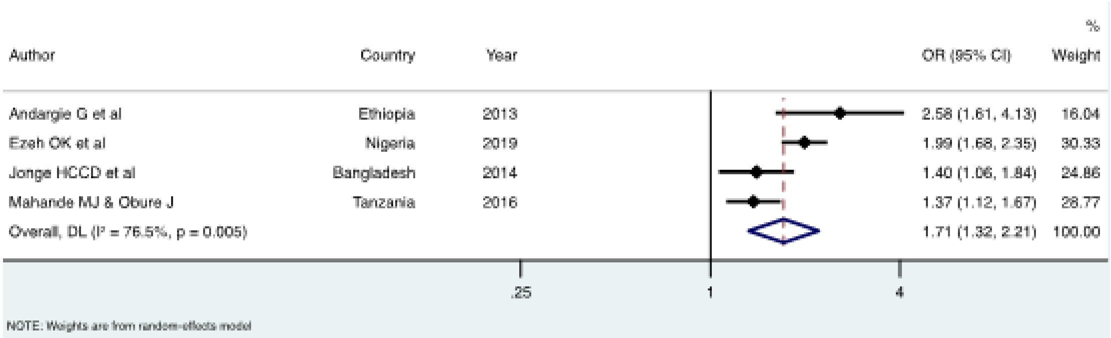

Supplementary Figure S3: Summary effect of short birth interval on perinatal mortality for low- and lower middle-income countries, January 2000 to January 2022

(a)

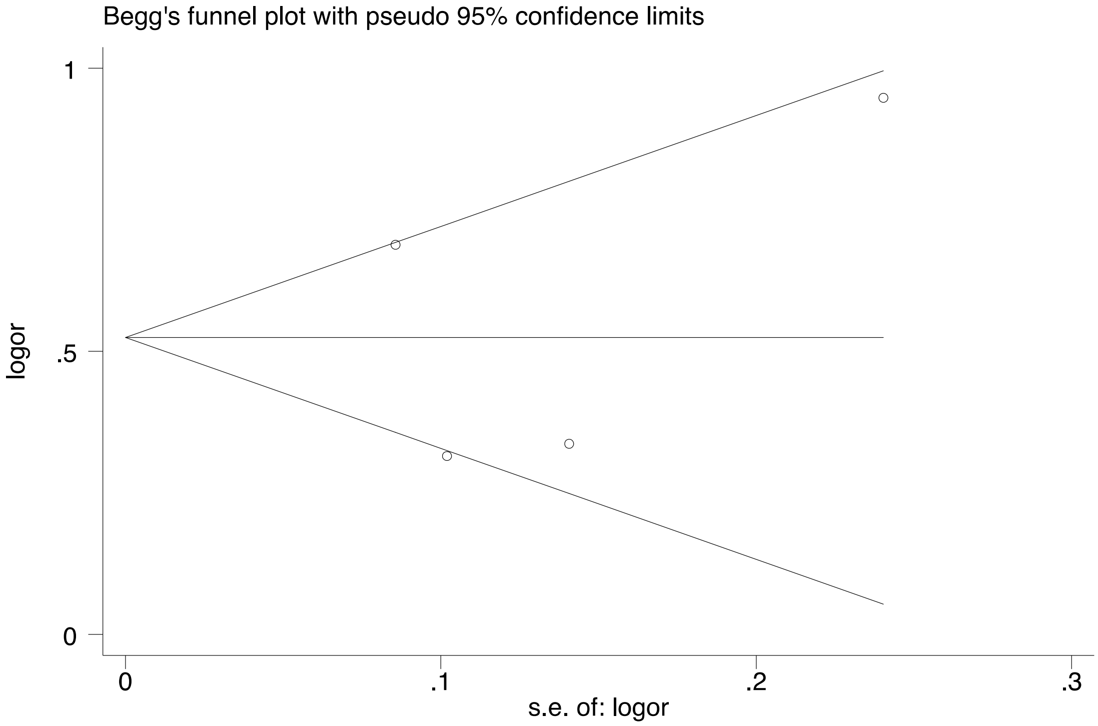

Supplementary Figure S3a: Evidence of publication bias in studies on perinatal mortality

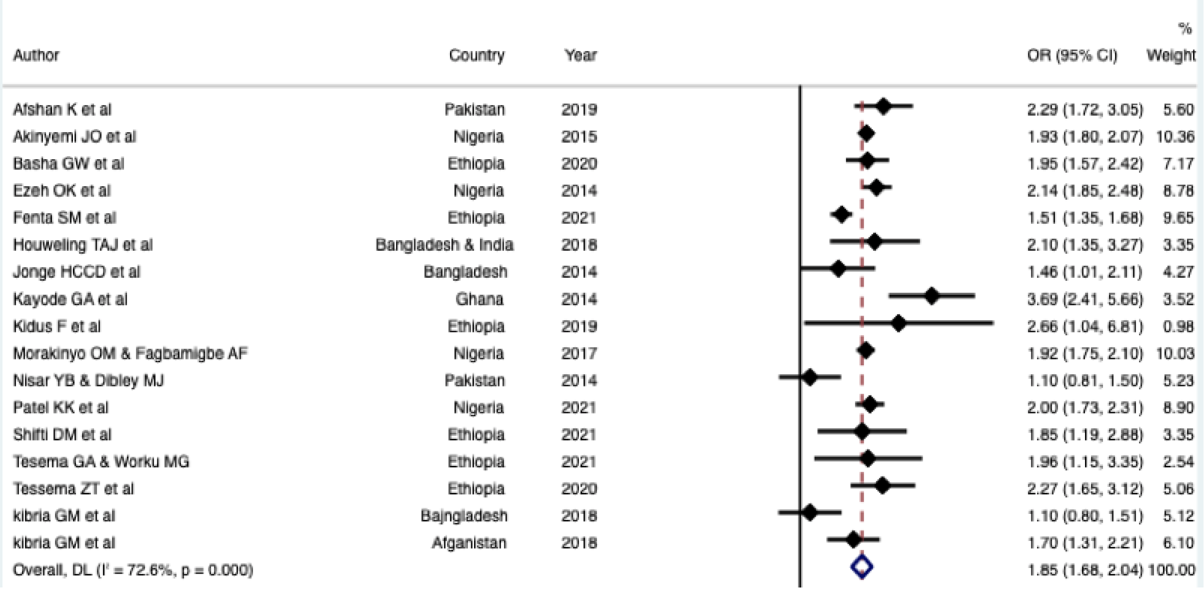

Supplementary Figure S4: Summary effect of short birth interval on neonatal mortality for low- and lower middle-income countries, January 2000 to January 2022

(a)

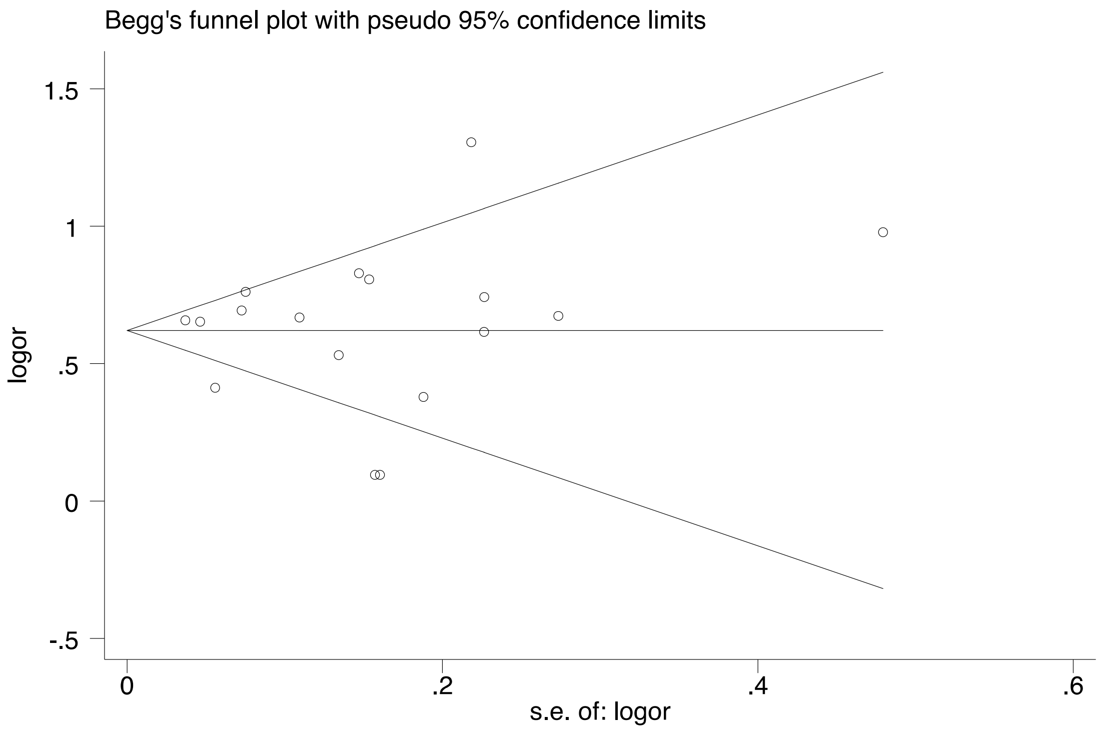

(b)

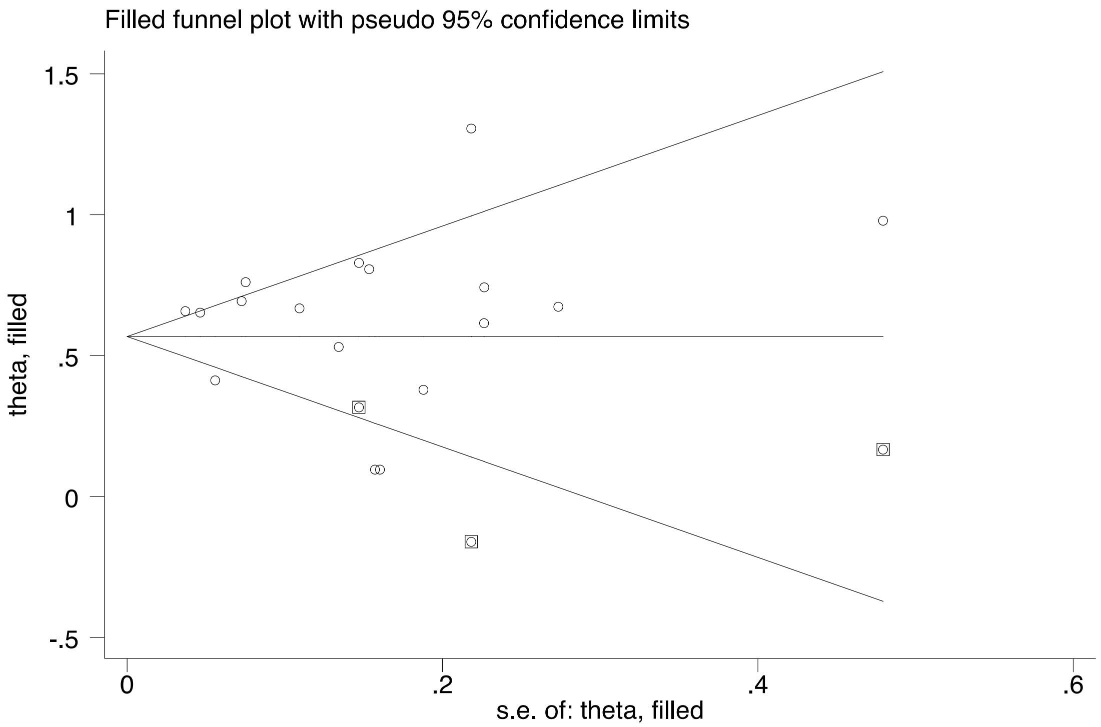

Supplementary Figure S4a: Evidence of publication bias in studies on neonatal mortality (a) without and (b) with trim and fill estimate

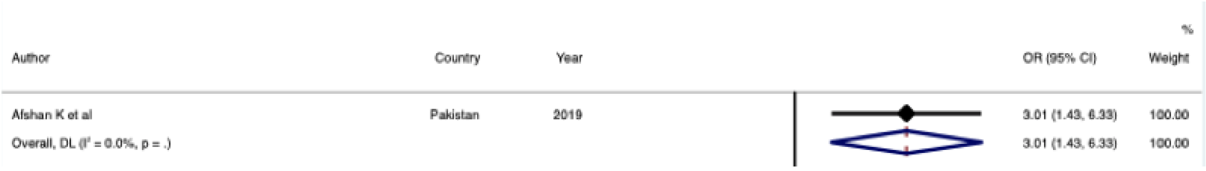

Supplementary Figure S5: Summary effect of short birth interval on post neonatal mortality for low- and lower middle-income countries, January 2000 to January 2022

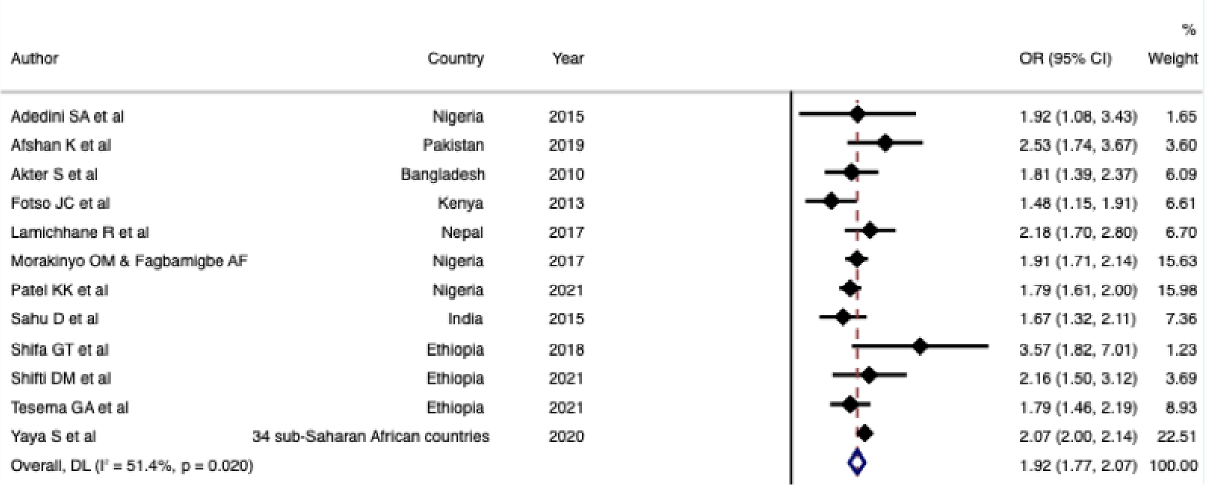

Supplementary Figure S6: Summary effect of short birth interval on infant mortality for low- and lower middle-income countries, January 2000 to January 2022

(a)

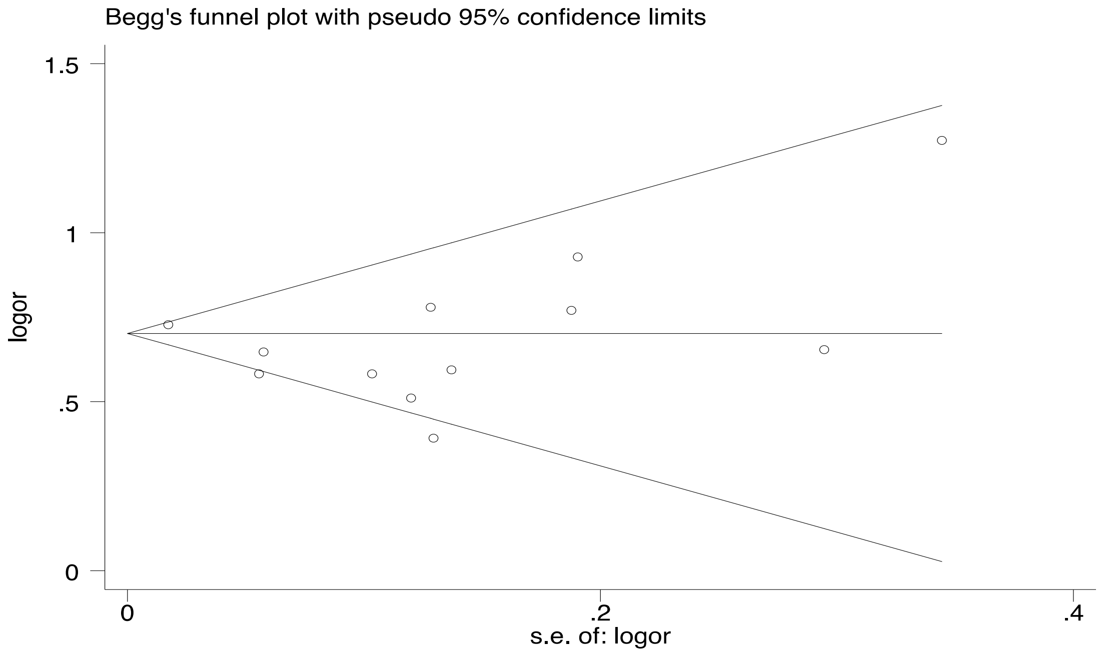

(b)

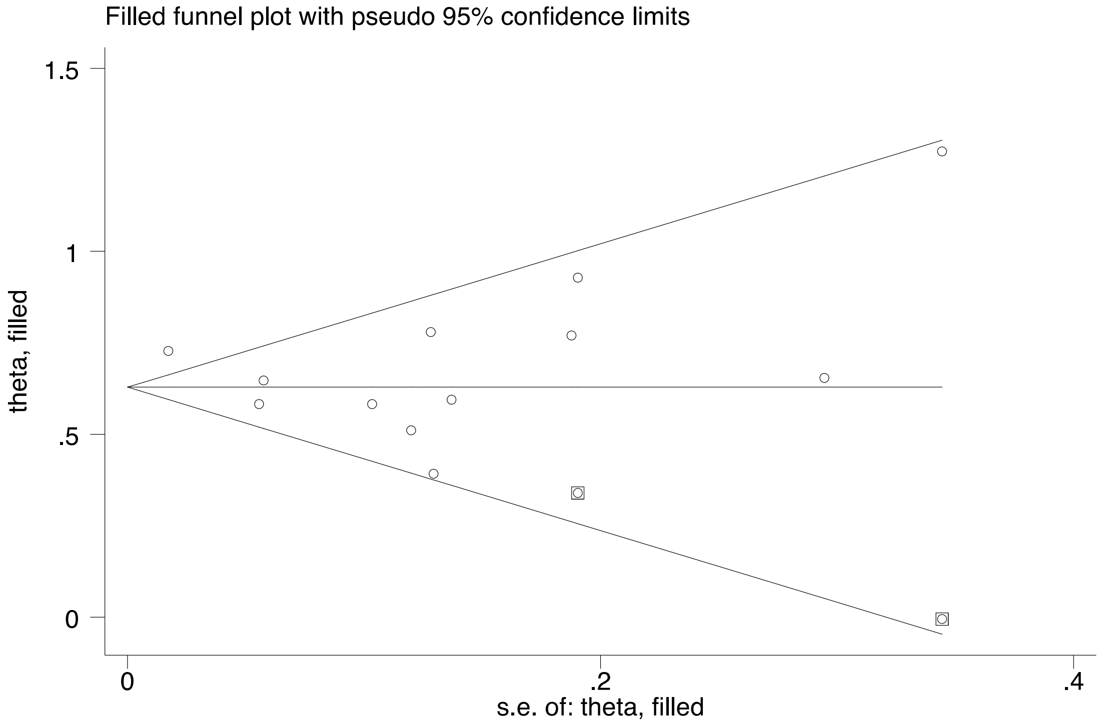

Supplementary Figure S6a: Evidence of publication bias in studies on infant mortality (a) without and (b) with trim and fill estimate

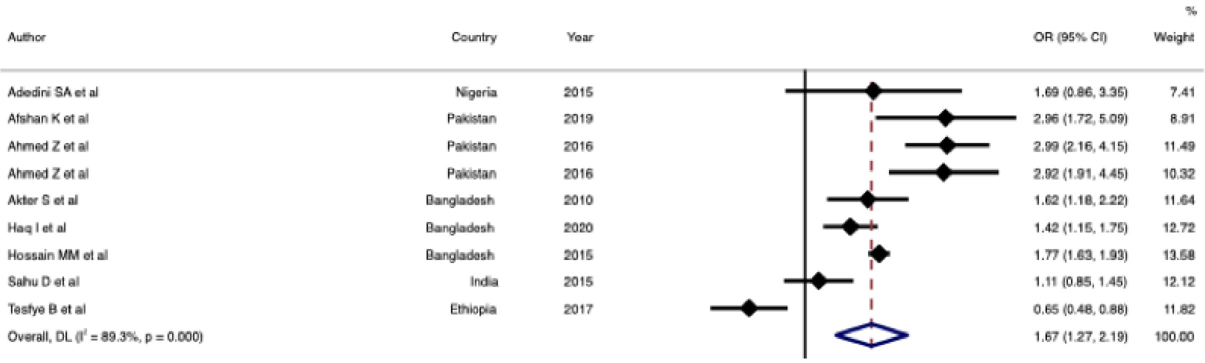

Supplementary Figure S7: Summary effect of short birth interval on child mortality for low- and lower middle-income countries, January 2000 to January 2022

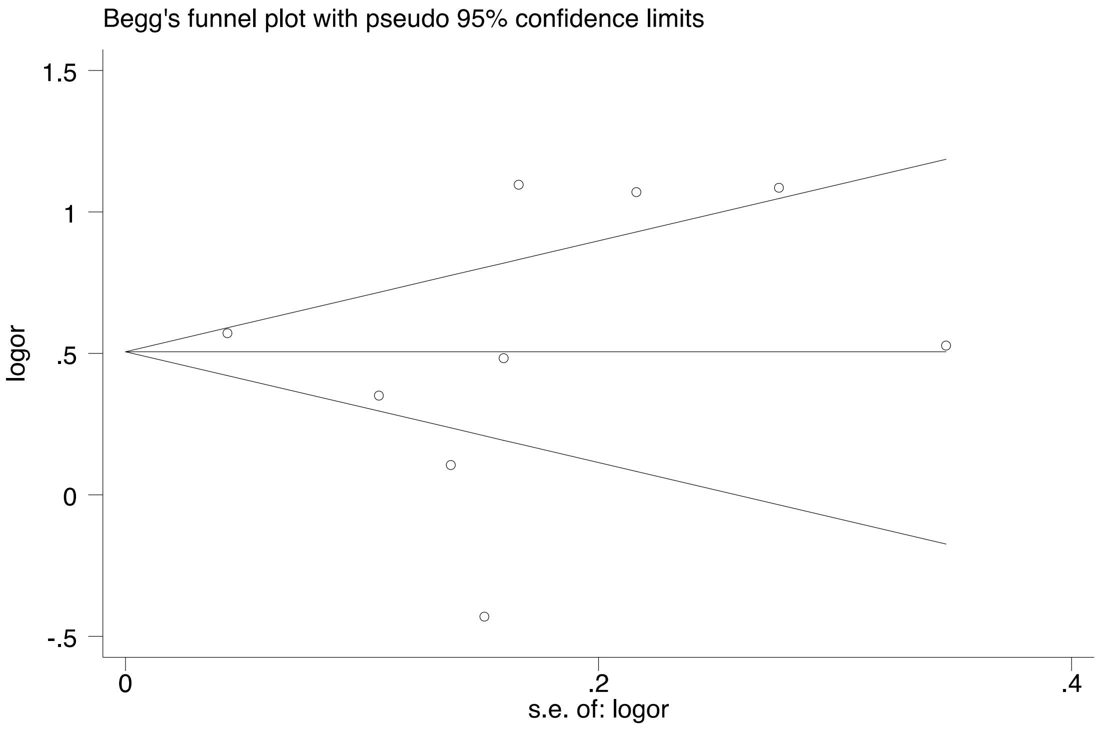

(b)

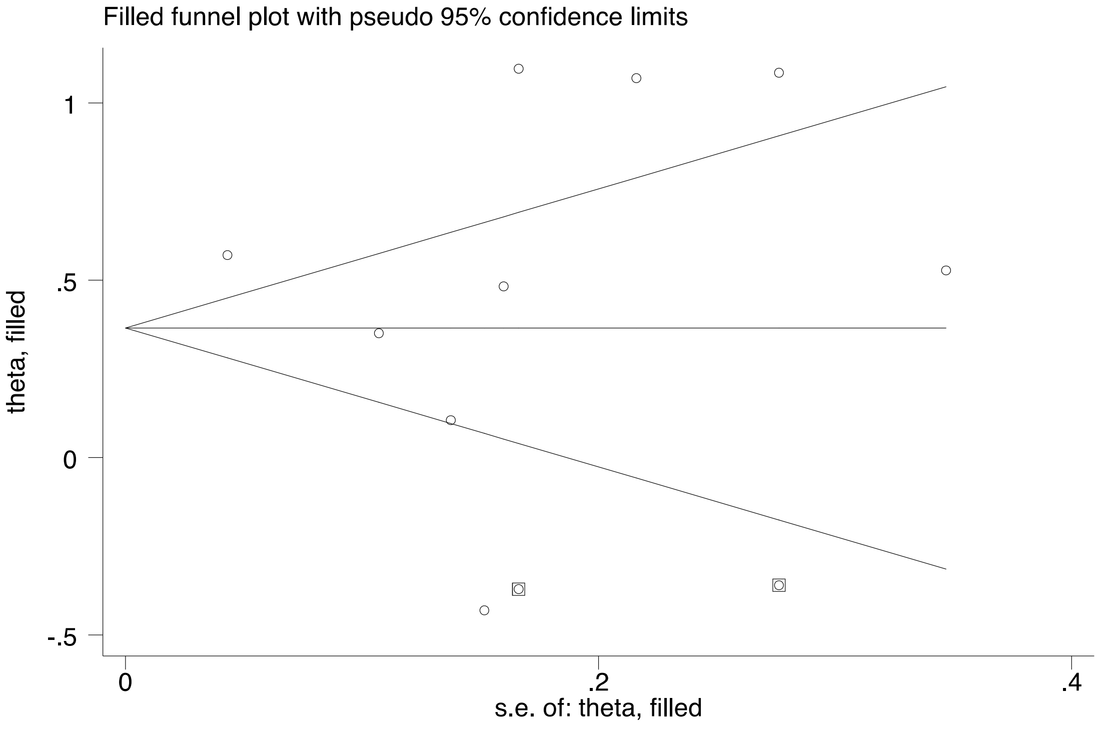

Supplementary Figure S7a: Evidence of publication bias in studies on Child mortality (a) without and (b) with trim and fill estimate

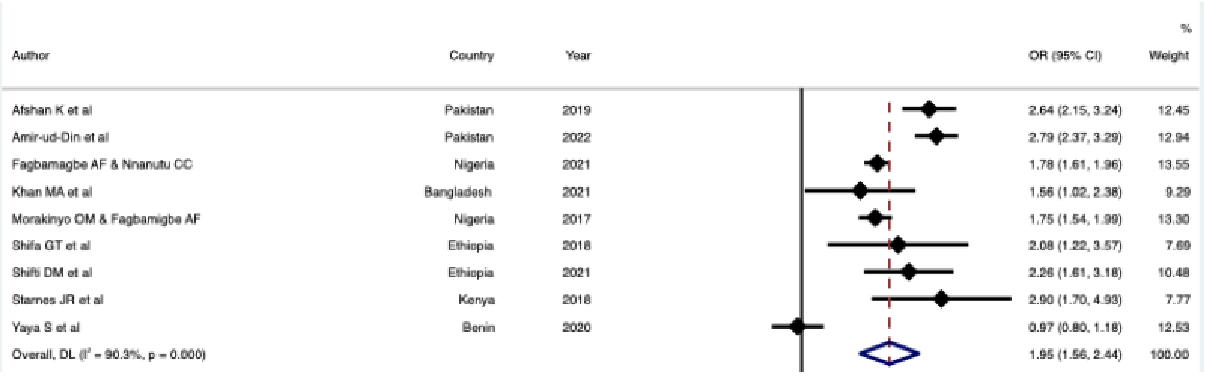

Supplementary Figure S8: Summary effect of short birth interval on under five mortality for low- and lower middle-income countries, January 2000 to January 2022

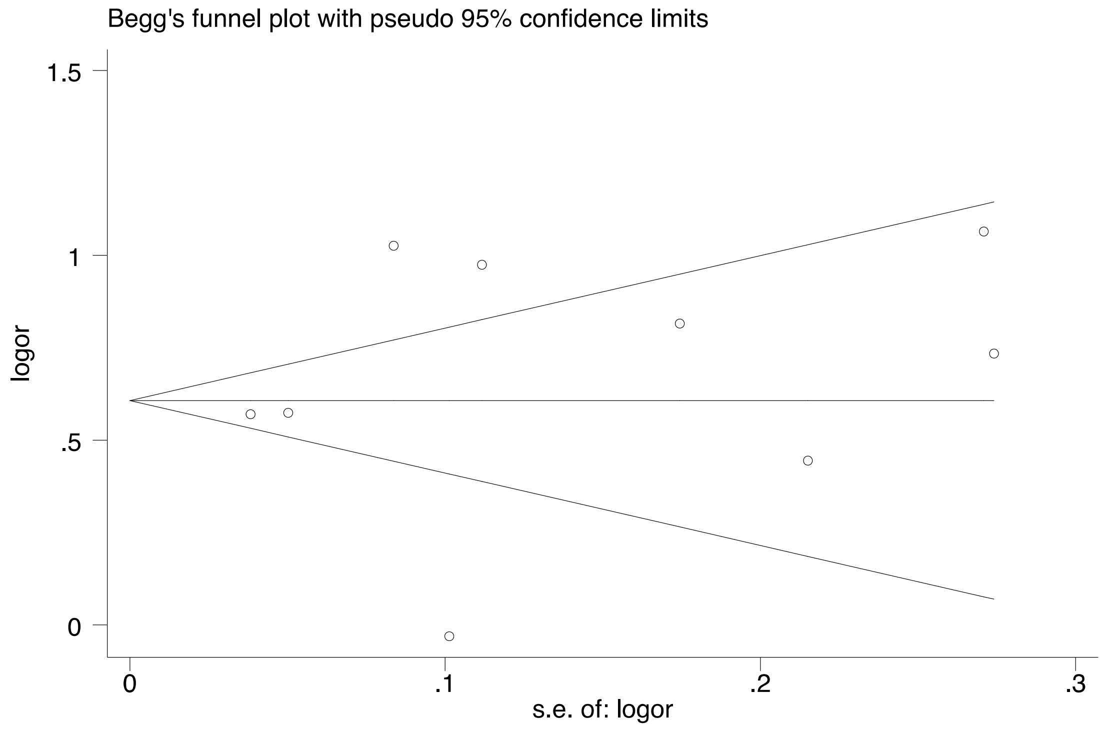

(b)

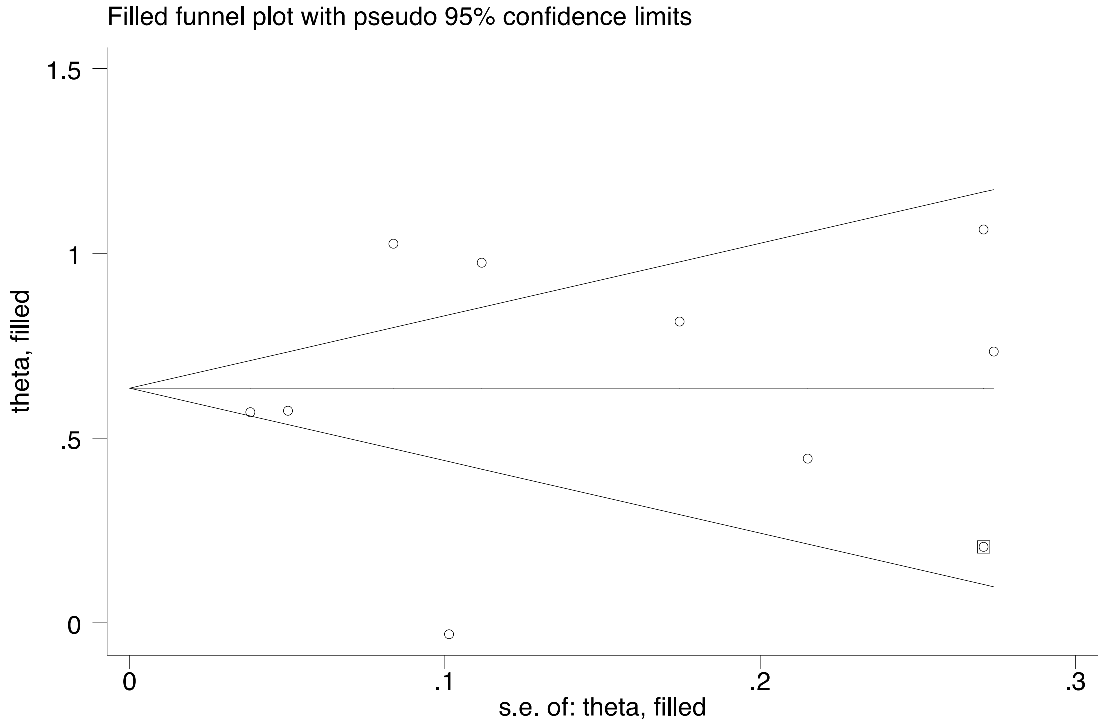

Supplementary Figure S8a: Evidence of publication bias in studies on under-five mortality (a) without and (b) with trim and fill estimate

Section/topic	#	Checklist item	Reported on page #
**TITLE**			
Title	1	Identify the report as a systematic review, meta-analysis, or both.	1
**ABSTRACT**			
Structured summary	2	Provide a structured summary including, as applicable: background; objectives; data sources; study eligibility criteria, participants, and interventions; study appraisal and synthesis methods; results; limitations; conclusions and implications of key findings; systematic review registration number.	1
**INTRODUCTION**			
Rationale	3	Describe the rationale for the review in the context of what is already known.	2
Objectives	4	Provide an explicit statement of questions being addressed with reference to participants, interventions, comparisons, outcomes, and study design (PICOS).	3
**METHODS**			
Protocol and registration	5	Indicate if a review protocol exists, if and where it can be accessed (e.g., Web address), and, if available, provide registration information including registration number.	NA
Eligibility criteria	6	Specify study characteristics (e.g., PICOS, length of follow-up) and report characteristics (e.g., years considered, language, publication status) used as criteria for eligibility, giving rationale.	4-7
Information sources	7	Describe all information sources (e.g., databases with dates of coverage, contact with study authors to identify additional studies) in the search and date last searched.	4-7
Search	8	Present full electronic search strategy for at least one database, including any limits used, such that it could be repeated.	4-7<br>Supplementary table 1-7
Study selection	9	State the process for selecting studies (i.e., screening, eligibility, included in systematic review, and, if applicable, included in the meta-analysis).	4

Data collection process	10	Describe method of data extraction from reports (e.g., piloted forms, independently, in duplicate) and any processes for obtaining and confirming data from investigators.	4
Data items	11	List and define all variables for which data were sought (e.g., PICOS, funding sources) and any assumptions and simplifications made.	4
Risk of bias in individual studies	12	Describe methods used for assessing risk of bias of individual studies (including specification of whether this was done at the study or outcome level), and how this information is to be used in any data synthesis.	5
Summary measures	13	State the principal summary measures (e.g., risk ratio, difference in means).	5
Synthesis of results	14	Describe the methods of handling data and combining results of studies, if done, including measures of consistency (e.g., I^2) for each meta-analysis.	5

**Section/topic**	**#**	**Checklist item**	**Reported on page #**
Risk of bias across studies	15	Specify any assessment of risk of bias that may affect the cumulative evidence (e.g., publication bias, selective reporting within studies).	8-9
Additional analyses	16	Describe methods of additional analyses (e.g., sensitivity or subgroup analyses, meta-regression), if done, indicating which were pre-specified.	8-9
**RESULTS**			
Study selection	17	Give numbers of studies screened, assessed for eligibility, and included in the review, with reasons for exclusions at each stage, ideally with a flow diagram.	8-9
Study characteristics	18	For each study, present characteristics for which data were extracted (e.g., study size, PICOS, follow-up period) and provide the citations.	8*9
Risk of bias within studies	19	Present data on risk of bias of each study and, if available, any outcome level assessment (see item 12).	8-9
Results of individual studies	20	For all outcomes considered (benefits or harms), present, for each study: (a) simple summary data for each intervention group (b) effect estimates and confidence intervals, ideally with a forest plot.	10, Supplementary Tables
Synthesis of results	21	Present results of each meta-analysis done, including confidence intervals and measures of consistency.	6

Risk of bias across studies	22	Present results of any assessment of risk of bias across studies (see Item 15).	6
Additional analysis	23	Give results of additional analyses, if done (e.g., sensitivity or subgroup analyses, meta-regression [see Item 16]).	8
**DISCUSSION**			
Summary of evidence	24	Summarize the main findings including the strength of evidence for each main outcome; consider their relevance to key groups (e.g., healthcare providers, users, and policy makers).	17
Limitations	25	Discuss limitations at study and outcome level (e.g., risk of bias), and at review-level (e.g., incomplete retrieval of identified research, reporting bias).	19-20
Conclusions	26	Provide a general interpretation of the results in the context of other evidence, and implications for future research.	20
**FUNDING**			
Funding	27	Describe sources of funding for the systematic review and other support (e.g., supply of data); role of funders for the systematic review.	20

From: Moher D, Liberati A, Tetzlaff J, Altman DG, The PRISMA Group (2009). Preferred Reporting Items for Systematic Reviews and Meta-Analyses: The PRISMA Statement. PLoS Med 6(6): e1000097. doi:10.1371/journal.pmed1000097

For more information, visit: **www.prisma-statement.org**.

References

1. Morakinyo OM, Fagbamigbe AF. Neonatal, infant and under-five mortalities in Nigeria: An examination of trends and drivers (2003-2013). PLOS ONE. 2017 Aug 9;12(8):e0182990.

2. Mekonnen Dagne H, Takele Melku A, Abdurkadir Abdi A. Determinants of Stillbirth Among Deliveries Attended in Bale Zone Hospitals, Oromia Regional State, Southeast Ethiopia: A Case-Control Study. Int J Womens Health. 2021;13:51–60.

3. Akinyemi JO, Bamgboye EA, Ayeni O. Trends in neonatal mortality in Nigeria and effects of bio-demographic and maternal characteristics. BMC Pediatrics. 2015 Apr 9;15(1):36.

4. Akter S, Rahman JAMS, Rahman MM, Abedin S. The influence of birth spacing on child survival in Bangladesh: a life table approach. World Health Popul. 2010;12(1):42–56.

5. Lakew D, Tesfaye D, Mekonnen H. Determinants of stillbirth among women deliveries at Amhara region, Ethiopia. BMC Pregnancy and Childbirth. 2017 Nov 13;17(1):375.

6. Fenta SM, Biresaw HB, Fentaw KD. Risk factor of neonatal mortality in Ethiopia: multilevel analysis of 2016 Demographic and Health Survey. Tropical Medicine and Health. 2021 Feb 4;49(1):14.

7. Lamichhane R, Zhao Y, Paudel S, Adewuyi EO. Factors associated with infant mortality in Nepal: a comparative analysis of Nepal demographic and health surveys (NDHS) 2006 and 2011. BMC Public Health. 2017 Jan 10;17(1):53.

8. Andargie G, Berhane Y, Worku A, Kebede Y. Predictors of perinatal mortality in rural population of Northwest Ethiopia: a prospective longitudinal study. BMC Public Health. 2013 Feb 23;13(1):168.

9. de Jonge HC, Azad K, Seward N, Kuddus A, Shaha S, Beard J, et al. Determinants and consequences of short birth interval in rural Bangladesh: a cross-sectional study. BMC Pregnancy and Childbirth. 2014 Dec 24;14(1):427.

10. Ahmed Z, Kamal A, Kamal A. Determinants of child mortality in Pakistan: Cox proportional hazard model analysis for PDHS (2006-07) and PDHS (2012-13). Pakistan Paediatric Journal. 2016 Mar 1;40:47–53.

11. Ezeh OK, Uche-Nwachi EO, Abada UD, Agho KE. Community-and proximate-level factors associated with perinatal mortality in Nigeria: evidence from a nationwide household survey. BMC Public Health. 2019 Jun 24;19(1):811.

12. Houweling TAJ, van Klaveren D, Das S, Azad K, Tripathy P, Manandhar D, et al. A prediction model for neonatal mortality in low- and middle-income countries: an analysis of data from population surveillance sites in India, Nepal and Bangladesh. Int J Epidemiol. 2019 Feb 1;48(1):186–98.

13. Fotso JC, Cleland J, Mberu B, Mutua M, Elungata P. Birth spacing and child mortality: an analysis of prospective data from the Nairobi urban health and demographic surveillance system. J Biosoc Sci. 2013 Nov;45(6):779–98.

14. Starnes JR, Chamberlain L, Sutermaster S, Owuor M, Okoth V, Edman W, et al. Under-five mortality in the Rongo Sub-County of Migori County, Kenya: Experience of the Lwala Community Alliance 2007-2017 with evidence from a cross-sectional survey. PLOS ONE. 2018 Sep 7;13(9):e0203690.

15. Tesema GA, Seretew WS, Worku MG, Angaw DA. Trends of infant mortality and its determinants in Ethiopia: mixed-effect binary logistic regression and multivariate decomposition analysis. BMC Pregnancy and Childbirth. 2021 May 5;21(1):362.

16. Tessema ZT, Tesema GA. Incidence of neonatal mortality and its predictors among live births in Ethiopia: Gompertz gamma shared frailty model. Italian Journal of Pediatrics. 2020 Sep 21;46(1):138.

17. Hossain MdM, Mani KKC, Islam MdR. Prevalence and Determinants of the Gender Differentials Risk Factors of Child Deaths in Bangladesh: Evidence from the Bangladesh Demographic and Health Survey, 2011. Franco-Paredes C, editor. PLoS Negl Trop Dis. 2015 Mar 6;9(3):e0003616.

18. Patel KK, Prasad JB, Biradar RA. Trends in and determinants of neonatal and infant mortality in Nigeria based on Demographic and Health Survey data. Journal of Biosocial Science. 2021 Nov;53(6):924–34.

19. Khan MA, Khan N, Rahman O, Mustagir G, Hossain K, Islam R, et al. Trends and projections of under-5 mortality in Bangladesh including the effects of maternal high-risk fertility behaviours and use of healthcare services. PLOS ONE. 2021 Feb 4;16(2):e0246210.

20. Adedini SA, Odimegwu C, Imasiku ENS, Ononokpono DN, Ibisomi L. REGIONAL VARIATIONS IN INFANT AND CHILD MORTALITY IN NIGERIA: A MULTILEVEL ANALYSIS. J Biosoc Sci. 2015 Mar;47(2):165–87.

21. Basha GW, Woya AA, Tekile AK. Determinants of neonatal mortality in Ethiopia: an analysis of the 2016 Ethiopia Demographic and Health Survey. Afr Health Sci. 2020 Jun;20(2):715–23.

22. Afshan K, Narjis G, Qureshi IZ, Cappello M. Social determinants and causes of child mortality in Pakistan: Analysis of national demographic health surveys from 1990 to 2013. Journal of Paediatrics and Child Health. 2020;56(3):457–72.

23. Kidus F, Woldemichael K, Hiko D. Predictors of neonatal mortality in Assosa zone, Western Ethiopia: a matched case control study. BMC Pregnancy and Childbirth. 2019 Mar 29;19(1):108.

24. Ahmed Z, Kamal A, Kamal A. Statistical Analysis of Factors Affecting Child Mortality in Pakistan. J Coll Physicians Surg Pak. 2016 Jun;26(6):543–4.

25. Kayode GA, Ansah E, Agyepong IA, Amoakoh-Coleman M, Grobbee DE, Klipstein-Grobusch K. Individual and community determinants of neonatal mortality in Ghana: a multilevel analysis. BMC Pregnancy and Childbirth. 2014 May 12;14(1):165.

26. Sahu D, Nair S, Singh L, Gulati BK, Pandey A. Levels, trends & predictors of infant & child mortality among Scheduled Tribes in rural India. The Indian journal of medical research. 2015 May 1;141:709–19.

27. Tesema GA, Gezie LD, Nigatu SG. Spatial distribution of stillbirth and associated factors in Ethiopia: a spatial and multilevel analysis. BMJ Open. 2020 Oct;10(10):e034562.

28. Mahande MJ, Obure J. Effect of interpregnancy interval on adverse pregnancy outcomes in northern Tanzania: a registry-based retrospective cohort study. BMC Pregnancy and Childbirth. 2016 Jun 7;16(1):140.

29. Tesfaye B, Atique S, Elias N, Dibaba L, Shabbir S-A, Kebede M. Determinants and development of a web-based child mortality prediction model in resource-limited settings: A data mining approach. Comput Methods Programs Biomed. 2017 Mar;140:45–51.

30. Ezeh OK, Agho KE, Dibley MJ, Hall J, Page AN. Determinants of neonatal mortality in Nigeria: evidence from the 2008 demographic and health survey. BMC Public Health. 2014 May 29;14(1):521.

31. Nisar YB, Dibley MJ. Determinants of neonatal mortality in Pakistan: secondary analysis of Pakistan Demographic and Health Survey 2006–07. BMC Public Health. 2014 Jun 28;14(1):663.

32. Shifa GT, Ahmed AA, Yalew AW. Maternal and child characteristics and health practices affecting under-five mortality: A matched case control study in Gamo Gofa Zone, Southern Ethiopia. PLOS ONE. 2018 Aug 15;13(8):e0202124.

33. Tesema GA, Worku MG. Individual-and community-level determinants of neonatal mortality in the emerging regions of Ethiopia: a multilevel mixed-effect analysis. BMC Pregnancy and Childbirth. 2021 Jan 6;21(1):12.

34. Haq I, Alam M, Islam A, Rahman M, Latif A, Methun MIH, et al. Influence of sociodemographic factors on child mortality in Bangladesh: a multivariate analysis. J Public Health (Berl) [Internet]. 2020 Oct 10 [cited 2021 Dec 6]; Available from: https://doi.org/10.1007/s10389-020-01378-w

35. Amir-ud-Din R, Mahmood HZ, Abbas F, Muzammil M, Kumar R, Pongpanich S. Association of breast feeding and birth interval with child mortality in Pakistan: a cross-sectional study using nationally representative Demographic and Health Survey data. BMJ Open. 2022 Jan 1;12(1):e053196.

36. Fagbamigbe A, Nnanatu C. Modelling the Spatial Distribution and the Factors Associated with Under-Five Mortality in Nigeria. Spatial Demography. 2021 Mar 29;

37. Shifti DM, Chojenta C, Holliday E, Loxton D. Effects of short birth interval on neonatal, infant and under-five child mortality in Ethiopia: a nationally representative observational study using inverse probability of treatment weighting. BMJ Open. 2021 Aug 1;11(8):e047892.

38. Yaya S, Ahinkorah BO, Ameyaw EK, Seidu A-A, Darteh EKM, Adjei NK. Proximate and socio-economic determinants of under-five mortality in Benin, 2017/2018. BMJ Glob Health. 2020 Aug;5(8):e002761.

39. Kibria GMA, Khanam R, Mitra DK, Mahmud A, Begum N, Moin SMI, et al. Rates and determinants of neonatal mortality in two rural sub-districts of Sylhet, Bangladesh. PLOS ONE. 2018 Nov 21;13(11):e0206795.

40. Kibria GMA, Burrowes V, Choudhury A, Sharmeen A, Ghosh S, Mahmud A, et al. Determinants of early neonatal mortality in Afghanistan: an analysis of the Demographic and Health Survey 2015. Global Health. 2018 Dec;14(1):47.

41. Yaya S, Uthman OA, Ekholuenetale M, Bishwajit G, Adjiwanou V. Effects of birth spacing on adverse childhood health outcomes: evidence from 34 countries in sub-Saharan Africa. The Journal of Maternal-Fetal & Neonatal Medicine. 2020 Oct 17;33(20):3501–8.
